# Machine Learning Approaches for Optimizing Drug Combinations in Neurodegenerative Diseases: A Brief Review

**DOI:** 10.1021/acsomega.5c07349

**Published:** 2025-11-30

**Authors:** Yawei Ma, Haijun Tian, Wenguang Xiao, Youfu Ma, Houlin Su, Li Zhu, Yu Jiang, Li Ge, Yan Li, Mingqing Yuan, Xu Liu

**Affiliations:** † Guangxi Key Laboratory of Special Biomedicine, School of Medicine, 12664 Guangxi University, Nanning 530004, China; ‡ Department of Medicinal Chemistry, School of Pharmacy, Fudan University, Shanghai 201203, China

## Abstract

As the global population ages, the prevalence of neurodegenerative diseases (NDDs)—including Alzheimer’s disease, Parkinson’s disease, Huntington’s disease, Multisystem Atrophy (multiple system atrophy), and amyotrophic lateral sclerosis—continues to rise, largely driven by environmental, metabolic, and lifestyle risk factors. Advances in computational technologies, particularly machine learning (ML) and deep learning, are reshaping research in this field. This review summarizes the major features of these diseases and emphasizes the role of ML in drug discovery, virtual screening, drug repurposing, and drug combination optimization. Representative approaches include support vector machines for classification, convolutional neural networks|convolutional neural network for imaging analysis, recurrent neural networks for temporal biomedical data, and transformers for multimodal integration. These methods highlight the potential of computational strategies to improve therapeutic development. In addition, the review underscores the substantial incidence rates and socioeconomic burden of these conditions, which have made them focal points for algorithmic innovation. With research evolving rapidly, the development of AI-driven approaches is expected to enable more effective, targeted interventions and improve patient outcomes. This Perspective provides a concise overview of current progress and identifies promising future directions in the fight against NDDs.

## Graphical abstract

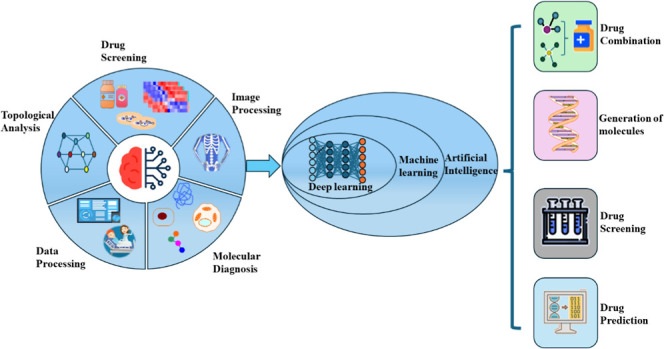

## 1 Introduction

Neurodegenerative diseases (NDDs), characterized by progressive neuronal degeneration, represent a growing global health concern, significantly impacting motor, cognitive, and behavioral functions.^1^ Major NDDs include Alzheimer’s disease (AD), Parkinson’s disease (PD), Amyotrophic lateral sclerosis (ALS), Dementia with Lewy Bodies (DLB), Multiple system atrophy (MSA), and Huntington’s disease (HD), each exhibiting distinct pathological features and clinical manifestations. The insidious onset of these conditions is often presented after substantial neuronal loss. Current pharmacological interventions, including monoamine oxidase B (MAO-B) inhibitors, COMT inhibitors, and dopamine agonists, primarily offer symptomatic relief without halting disease progression.^2–4^ The complexity of NDDs’ pathogenesis, involving multiple biological pathways and gene–environment interactions, necessitates innovative therapeutic strategies.

In parallel with these clinical challenges, large-scale epidemiological analyses have quantified the global burden of neurological disorders, Based on epidemiological data from the Global Burden of Disease study,^5^ mortality, prevalence, years lived with disability, years of life lost, and disability-adjusted life-years (DALYs) were quantified for 37 neurological conditions across 204 countries and territories from 1990 to 2021. The findings revealed that neurological disorders had become the leading cause of DALYs globally in 2021, affecting more than 3.4 billion individuals, with stroke, AD and other dementias, and diabetic neuropathy identified as major contributors. According to projections by the World Health Organization, NDDs are expected to surpass cardiovascular diseases as the leading cause of motor dysfunction within the next two decades and become the second leading cause of death after cancer.^6^ These quantitative estimates underscore the profound impact of neurological disorders on global health and socioeconomic systems.

NDDs represent multifactorial disorders characterized by complex etiologies involving diverse genetic, molecular, and environmental mechanisms. Monotherapies often fail to address this biological heterogeneity, frequently requiring high dosages that increase adverse effects and contribute to drug resistance. The estimated investment to bring a new drug to market ranges from US$314 million to US$2.8 billion, with a development timeline of approximately 10 to 15 years,^7^ In this context, both traditional approaches and machine learning (ML)-based methods have been widely applied in NDD research and clinical practice to support diagnosis, prognosis, and drug development. Traditional approaches typically rely on clinical scales, established biomarkers, and rule-based statistical models, offering high interpretability and clinical validation. In contrast, ML approaches—including classical algorithms and deep learning (DL) models—leverage multimodal data to uncover complex patterns and predict outcomes, though they often require large data sets and extensive validation. Table 1 summarizes these commonly used methods, highlighting their typical applications as well as their respective strengths and limitations.

**Table 1:** Comparison of Common Methods in NDD Research

method category	typical methods/algorithms	main applications	advantages	limitations
**traditional approaches**	MMSE, MoCA, ACE, UPDRS, ALSFRS-R	clinical screening and functional assessment	high interpretability; well-validated; clinically feasible	low sensitivity to early stage disease; limited ability to handle large-scale data
	MRI, PET, CSF/blood biomarkers	diagnosis and prognosis prediction	reliable and standardized	high acquisition cost; limited ability to detect complex patterns
	rule-based/statistical models (Kaplan–Meier, Cox regression, logistic regression scoring systems)	prognostic analysis, risk prediction	transparent; reproducible	poor handling of nonlinear or multimodal data
**machine learning approaches**	SVM, RF, LR, k-NN, NB	classification of clinical or omics features	can model complex relationships; integrates multiple features	limited performance on small data sets; low interpretability
	CNN, RNN/LSTM, GNN, autoencoder, GAN	image analysis, temporal data, drug combination prediction	handles large-scale and multimodal data; automatic feature extraction	high data requirements; “black-box” nature; limited clinical validation
	DeepSynergy, MatchMaker, graph autoencoder (GAE)	drug combination prediction	can uncover potential drug–drug interactions	complex models require external validation

Taken together, this comparison illustrates that traditional methods provide a reliable, well-validated framework for clinical evaluation, whereas ML approaches offer enhanced capability to capture complex, multidimensional patterns that may be invisible to conventional analyses. Such a complementary perspective underscores the value of integrating both approaches in NDD research and therapeutic development. Building on this methodological foundation, ML has emerged as a powerful tool for developing personalized combination therapies. By integrating data from clinical trials, genetic profiles, and patient outcomes, the ML can identify novel treatment patterns and optimize therapeutic efficacy while minimizing adverse effects. For example, PD treatments such as levodopa-carbidopa may induce hepatic and gastrointestinal complications, whereas dopamine agonists have been associated with balance impairments and sleep disorders,^8^ These challenges underscore the increasing emphasis on combination therapies, which aim to simultaneously target multiple disease pathways to enhance efficacy and reduce side effects.

In parallel, ML has emerged as a powerful computational paradigm capable of addressing the multifaceted demands of NDD treatment. ML algorithms offer robust solutions for early diagnosis, disease classification, and progression prediction while also driving innovation in drug discovery and repositioning. The integration of ML into high-throughput screening and virtual screening (VS) workflows—particularly through Computer-Aided Drug Design (CADD)—has significantly accelerated the identification of promising candidates. These advances collectively position ML as a cornerstone technology in the development of effective, cost-efficient, and personalized therapeutic strategies for NDDs.

Fortunately, given how quickly artificial intelligence (AI) is developing, there are chances to remedy this problem. The integration of multimodal data has demonstrated increasing value in the study of NDDs. By combining genomics, imaging, clinical records, and other biomarkers, multimodal approaches can capture the complex characteristics of diseases more comprehensively, thereby improving the predictive accuracy and model generalizability. The rapid expansion of ML has been driven by technological advancements like big data and cloud computing since the turn of the twenty-first century. ML’s performance and range of applications have greatly improved because of emerging technologies like DL and reinforcement learning. This rapid transformation in ML technology has sparked intense interest in harnessing these techniques to effectively mine transcriptomics,^9^ structural,^10^ and clinical data.^11^ As a type of AI algorithm and one of the fundamental paradigms of AI, ML is extremely valuable when it comes to using statistical methods to reveal latent connections and patterns in data. These methods greatly enhance the multisource data set’s extensive analytical capabilities. Through the creative application of these techniques, scientists can more quickly design therapeutic interventions by effectively screening and assessing the efficacy of the currently available medications in the treatment of neurodegenerative illnesses. This procedure not only highlights ML’s great potential in biomedical research but also offers fresh approaches to solving challenging health issues.^12^

AI and ML methodologies possess formidable capabilities in integrating diverse types of data and have widespread applications across various domains, including data mining, model building, and image recognition.^13^ Furthermore, numerous studies have demonstrated the superiority of utilizing ML for early diagnosis, classification, identification, and progression prediction of NDDs.^14–16^ Additionally, there is a growing consensus among researchers on employing ML tools in drug discovery and clinical trials.^17,18^ AI and computational technologies are being harnessed to screen and analyze millions of compounds within libraries to identify the most promising drug candidates. CADD has gained significant importance and attention in recent years,^19^ as it offers a powerful approach to streamline the drug discovery process.

Current treatments for AD include acetylcholinesterase inhibitors, which increase acetylcholine levels in the brain and enhance synaptic transmission, β-amyloid antibodies, and NMDA antagonists, which regulate glutamate activity by antagonizing the NMDA receptor, thereby relieving symptoms.^20^ These methods, however, are not very effective in treating AD patients. Thus, to improve patient symptoms, it is imperative to develop novel therapeutic approaches and medications that can postpone or prevent the development of the disease and slow its progression.

In this review, AD and PD are discussed in greater detail due to the abundance of research studies and available data sets. HD and ALS are also reviewed with reference to recent ML studies. MSA is included as an underexplored condition with limited computational literature to highlight important research gaps and future opportunities.

## 2 Overview of Neurodegenerative Diseases

NDDs characterized by progressive neuronal loss in the central nervous system or peripheral nervous system represent a significant global health burden affecting millions worldwide. The irreversible nature of neuronal degeneration, coupled with the inability of terminally differentiated neurons to self-renewal, leads to the breakdown of neural networks and subsequent impairment of cognitive, sensory, motor, and behavioral functions. Current understanding of NDD pathogenesis, as reviewed by Wilson et al.,^21^ reveals multiple interconnected pathological mechanisms: (1) pathological protein aggregation, (2) synaptic transmission dysfunction, (3) protein homeostasis imbalance, (4) cytoskeletal abnormalities, (5) metabolic pathway alterations, (6) nucleic acid defects, (7) chronic inflammation, and (8) neuronal apoptosis. These processes collectively drive neurodegeneration through a “multistrike process”, where protein misfolding and aggregation not only induce toxicity but also create spatial imbalances in protein distribution, leading to functional deficits. This complex interplay between gain-of-toxicity and loss-of-function mechanisms underscores the necessity for multitargeted therapeutic strategies addressing various pathogenic aspects to effectively modify disease progression. To better contextualize how various ML paradigms contribute to different stages of drug research, we classify common algorithms into four categories based on their learning strategy, core principles, and typical applications, as shown in Table 2.

**Table 2:** Core Characteristics of Major NDDs

disease	pathological hallmarks	clinical features	key molecular targets/mutations	machine learning applications	main medications
AD	Aβ plaques, tau tangles, synaptic loss	memory decline, cognitive impairment, behavioral changes	APOE4, APP, PSEN1, PSEN2	target discovery, transcriptomics-based repositioning, and imaging diagnosis	donepezil, rivastigmine, galantamine, memantine
PD	α-synuclein accumulation, dopaminergic neuron loss	tremor, bradykinesia, rigidity, sleep disturbance	SNCA, LRRK2, PARKIN	imaging classification, multitarget drug discovery, and combination prediction	levodopa, carbidopa, pramipexole, ropinirole, MAO-B inhibitors
ALS	motor neuron death, neuroinflammation, neuromuscular junction degeneration	muscle atrophy, dysphagia, respiratory failure	SOD1, C9orf72, TARDBP, FUS	survival prediction, biomarker identification, anti-inflammatory screening	riluzole, edaravone, sodium phenylbutyrate/taurursodiol
DLB	lewy body formation (α-syn aggregates) in neurons	fluctuating cognition, hallucinations, REM sleep disorder, parkinsonism	SNCA, GBA	imaging-assisted diagnosis, transcriptome classification, shared-pathway drug repurposing	donepezil, rivastigmine, memantine
HD	mHtt protein aggregation, transcriptional dysregulation	chorea, cognitive impairment, psychiatric symptoms	HTT (CAG repeat expansion)	symptom onset prediction, HTT-targeted screening, deep modeling of mHtt	tetrabenazine, deutetrabenazine
MSA	α-syn in oligodendrocytes, glial cytoplasmic inclusions	autonomic failure, cerebellar ataxia, rigidity	SNCA, COQ2	subtype classification, imaging analysis, pathway modeling in glial cells	fludrocortisone, midodrine, droxidopa

To more intuitively illustrate the “multistrike process” and the complex interplay of pathological mechanisms underlying NDDs, Figure 1 uses AD as an example to summarize the major proposed pathogenic hypotheses and their interactions. These include the amyloid hypothesis, tau protein hypothesis, glutamatergic excitotoxicity hypothesis, cholinergic hypothesis, metal ion imbalance hypothesis, abnormal autophagy hypothesis, inflammatory hypothesis, and the microbiota–gut–brain axis hypothesis. This integrative view underscores the need for multitarget therapeutic strategies in NDDs and offers a multidimensional framework to guide drug discovery and development.

**Figure 1:**
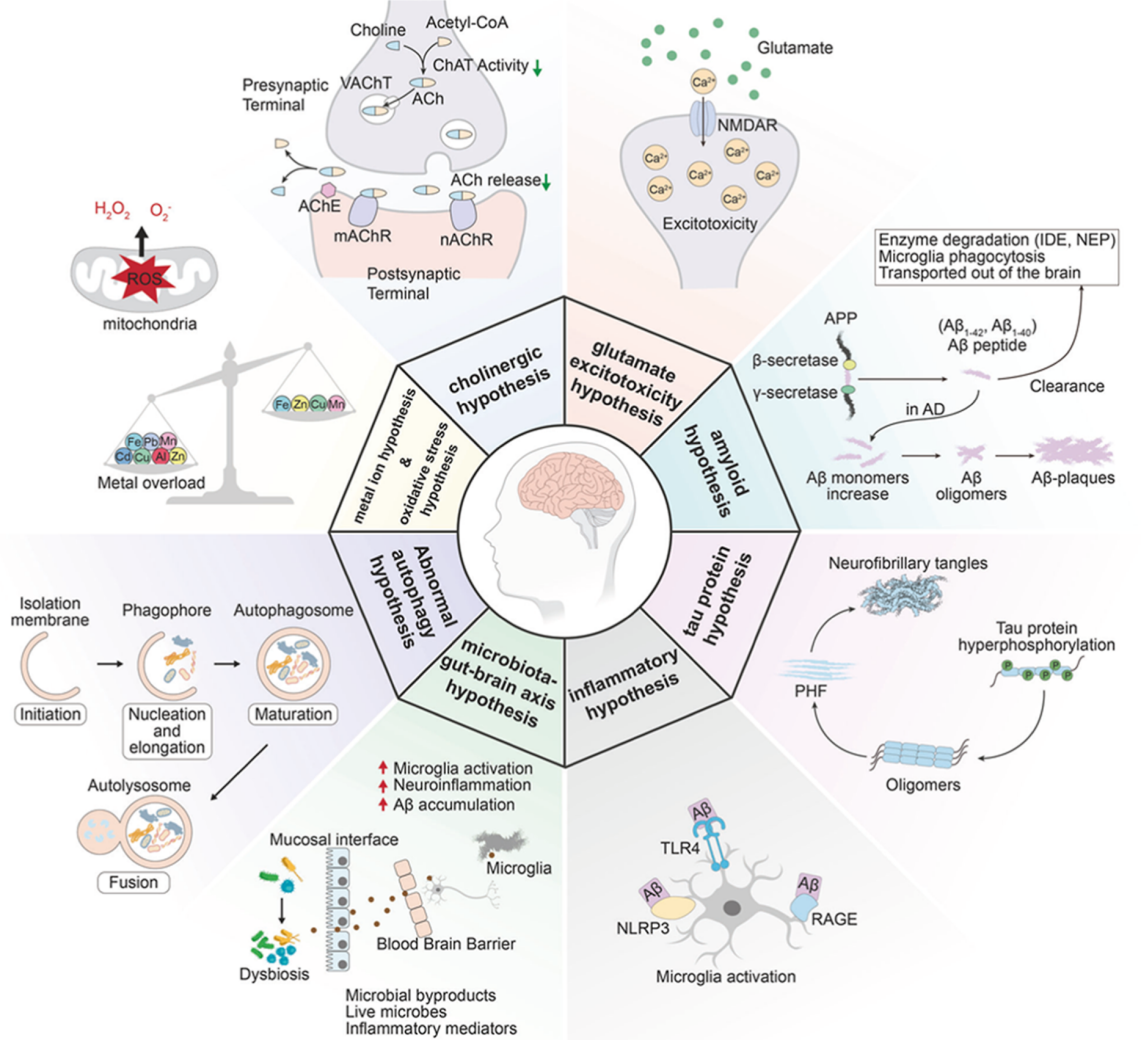
Diagram for the pathogenesis of AD. Reprinted from Zhang et al.,^22^ Signal Transduction and Targeted Therapy, 2024, 9, 211. Copyright 2024 The Authors. Published by Springer Nature under CC BY 4.0.

### 2.12.1 Alzheimer’s Disease

AD is the most prevalent form of dementia,^23^ characterized by progressive cognitive decline, memory loss, and behavioral changes.^24^ Its pathological hallmarks include amyloid-β (Aβ) plaque accumulation, hyperphosphorylated tau neurofibrillary tangles, and synaptic degeneration, particularly in the hippocampal and cortical regions. Genetic factors such as mutations in APP, PSEN1, and PSEN2,^25^ as well as the APOE4 allele,^26^ significantly increase disease risk. Current pharmacological treatments—cholinesterase inhibitors and NMDA receptor antagonists—provide only symptomatic relief.

Recent advances in ML have facilitated multiomics integration,^27^ enabling the identification of AD-related genes, biomarkers, and drug candidates. ML models are also used for image-based diagnosis, VS of compounds, and drug repurposing using transcriptomic data (e.g., CMap, DeepCE). This makes AD a leading use case for AI-driven NDDs research.

### 2.22.2 Parkinson’s Disease

PD is a movement disorder marked by bradykinesia,^28^ resting tremors, muscle rigidity, and postural instability. The core pathology involves the selective degeneration of dopaminergic neurons in the substantia nigra and intracellular accumulation of misfolded α-synuclein (forming Lewy bodies). These events cause a drastic reduction in dopamine levels, disrupting motor control and other cognitive functions.

Genetic mutations in SNCA, LRRK2, and PARKIN have been linked to familial PD forms. Although levodopa-based therapy remains standard, it often leads to side effects, and drug resistance. ML techniques such as support vector machines (SVM) (SVMs), convolutional neural networks (CNNs), and graph models are increasingly used for early diagnosis from imaging data, identification of novel targets (e.g., LRRK2, MAO-B), and synergy prediction in drug combinations. Computational approaches have enabled more precise modeling of dopaminergic pathways and patient subtypes.

### 2.32.3 Amyotrophic Lateral Sclerosis

ALS is a fatal motor neuron disease characterized by the progressive degeneration of upper and lower motor neurons,^29^ leading to muscle atrophy, weakness, dysphagia, and respiratory failure. Its pathological mechanisms involve glutamate excitotoxicity, neuroinflammation, and axonal transport disruption. Affected regions include the motor cortex, brainstem, and spinal cord.

Key genetic contributors include SOD1, TARDBP, FUS, and C9orf72, although many cases remain sporadic. Current treatments (e.g., Riluzole) offer minimal survival benefit. ML applications in ALS focus on survival prediction, gene expression analysis, and biomarker identification. Multimodal ML frameworks integrating clinical, imaging, and omics data are being explored for patient stratification and therapeutic targeting. Predictive models have also been used to assess drug efficacy and explore novel anti-inflammatory agents in silico.

### 2.42.4 Dementia with Lewy Bodies

DLB is the second most common form of neurodegenerative dementia after AD.^30^ It shares pathological similarities with PD, including the accumulation of α-synuclein aggregates into Lewy bodies within the cortical and subcortical neurons. Clinically, DLB is marked by fluctuating cognition, visual hallucinations, REM sleep behavior disorder, and Parkinsonian motor symptoms.

DLB pathogenesis also involves genetic associations such as mutations in SNCA, GBA, and genes regulating vesicle trafficking, and ML tools are used in DLB for differential diagnosis from AD or PD based on clinical profiles and imaging biomarkers. Emerging research leverages ML for the transcriptome-based classification and potential identification of repurposed drugs that act on shared α-synuclein pathways.

### 2.52.5 Huntington’s Disease

HD is a monogenic, autosomal dominant neurodegenerative disorder caused by CAG trinucleotide repeat expansions in the huntingtin (HTT) gene.^31^ The resulting mutant HTT (mHtt) protein disrupts neuronal function through misfolding, aggregation, and transcriptional dysregulation.

Symptoms typically begin in midadulthood and include choreiform involuntary movements, psychiatric disturbances, and progressive cognitive decline. Though diagnosis is confirmed via genetic testing, no disease-modifying therapy exists. ML is applied in HD research to analyze the relationship between CAG length and symptom onset, predict disease progression, and screen small molecules or antisense oligonucleotides targeting mHtt.^32^ DL models are also employed in the structural modeling of HTT-targeted therapies.

### 2.62.6 Multiple System Atrophy

MSA is a rare, rapidly progressive NDD featuring autonomic dysfunction,^33^ parkinsonism, and cerebellar ataxia. The pathological hallmark is the accumulation of misfolded α-synuclein in oligodendrocytes rather than neurons, forming glial cytoplasmic inclusions.

Affected brain regions include the basal ganglia, brainstem, and cerebellum. MSA pathogenesis involves neuroinflammation, mitochondrial dysfunction, and myelin dysregulation. ML research in MSA is still emerging but shows promise in subtype classification, autonomic symptom prediction, and connectivity-based imaging analysis. Integration of transcriptomic data with ML models may further clarify α-synuclein-related pathways unique to MSA versus those of PD.

## 3 Machine Learning and Its Application in Drug Research

ML has become an indispensable tool in modern drug discovery, particularly in addressing the complexity and heterogeneity of NDDs. With its ability to extract meaningful patterns from high-dimensional data, ML enables novel insights across various stages of the drug development pipeline. This section introduces core ML concepts and algorithm categories, followed by a detailed examination of their applications in drug research, including target identification, VS, drug repositioning, and combination therapy prediction. Furthermore, we explore the challenges of model evaluation, performance metrics, and future opportunities in interpretable and generalizable ML frameworks.

### 3.1 Brief Overview of Machine Learning

ML, originally defined by Arthur Samuel in 1959 as “the field of study that gives computers the ability to learn without being explicitly programmed,” has become integral to modern biomedical research.^34^ Its power to identify patterns and build predictive models from large-scale,^35^ high-dimensional data makes it particularly valuable in NDDs drug discovery, where data complexity and disease heterogeneity pose major challenges.

ML algorithms are broadly categorized into supervised, unsupervised, and generative (DL) paradigms.^36^ Supervised learning utilizes labeled data to perform classification or regression tasks and is widely applied in target identification, compound screening, and toxicity prediction.^37,38^ Unsupervised learning, by contrast, focuses on discovering intrinsic data structures useful for clustering compounds or reducing feature dimensionality. Generative models such as generative adversarial networks (GANs) and VAEs have recently gained attention for novel molecule generation and simulation of transcriptomic profiles.

ML and DL represent two major subfields of AI that share the overarching objective of deriving predictive insights from data. Both approaches are inherently data-driven, emphasize generalization, and have been applied extensively across domains such as medical diagnosis, image analysis, and natural language processing. Despite these similarities, notable distinctions exist. Traditional ML algorithms, such as SVMs, DTs, and RFs, typically rely on handcrafted feature engineering and demonstrate robust performance on small to medium-sized data sets. In contrast, DL leverages hierarchical neural network architectures to automatically learn feature representations, thereby reducing the reliance on manual intervention. However, this advantage comes at the cost of requiring large-scale data sets and substantial computational resources (e.g., GPU/TPU acceleration). Furthermore, traditional ML models are generally more interpretable, whereas DL approaches are often regarded as “black-box” systems, which may limit their transparency in clinical decision-making.^39^

To highlight these distinctions more clearly, Table 3 provides a comparative overview of traditional ML and DL across several key dimensions, including data requirements, feature extraction, computational demand, interpretability, and application domains. This comparative framework not only underscores their complementary strengths but also illustrates why the choice between ML and DL often depends on the scale, complexity, and specific goals of a given biomedical research problem.

**Table 3:** Comparison between Traditional ML and DL

aspect	traditional machine learning	deep learning
**data dependency**	perform effectively with small to medium data sets	requires large-scale data sets for optimal performance
**feature extraction**	reply to manual feature engineering	learns hierarchical representations automatically
**model complexity**	relatively simple (e.g., SVM, decision trees)	highly complex, multilayer neural networks
**computational demand**	modest computational requirements	high computational cost; strong reliance on GPUs/TPUs
**interpretability**	models are relatively transparent and interpretable	often considered opaque or “black-box”
**application scenarios**	suitable for tasks with well-defined features and limited data	excels in complex domains such as image, speech, and NLP tasks

Rather than detailing each algorithm, Table 4 summarizes the major ML/DL algorithm families, their core principles, and their typical applications in drug development. Abbreviations of ML algorithms: LR- Logistic Regression,^40^ SVM- SVM, DT-decision trees, RF- random forests,^41^ NB- naïve bayes,^42^ XGBoost -eXtreme gradient boosting,^43^ KMA- K-means algorithm,^44^ KNN-K-nearest neighbors, AE-autoencoders,^45^ ANN-artificial neural networks,^46^ DNN-deep neural networks, CNN-convolutional neural networks,^47^ RNN- recurrent neural networks, DBN-deep belief networks, GAN-generative adversarial networks­(Figure 2), Among these, CNN is particularly suitable for processing image data (Figure 3), In the following sections, we will explore how these algorithms are applied to VS, drug repositioning, and drug combination prediction in the context of NDDs.

**Table 4:** Common ML/DL Algorithms and Applications in Drug Discovery

algorithm type	key algorithms	main features	application in drug research
supervised learning	LR, SVM, RF, DNN, XGBoost	requires labeled data; high accuracy in prediction	drug–target interaction prediction,^48^ QSAR modeling, toxicity classification,^49^ drug discovery^40^
unsupervised learning	K-means, AE, DBN	pattern discovery, dimensionality reduction	compound clustering, latent feature extraction^45^
deep learning/generative models	CNN, RNN, GAN, VAE	feature abstraction, sequence and image processing, data generation	image-based screening,^50^ SMILES sequence modeling, novel drug generation
ensemble & hybrid models	RF, XGBoost, NB-SVM	combines multiple weak learners, reduces overfitting	target fishing, multitask prediction, polypharmacology^51^

**Figure 2:**
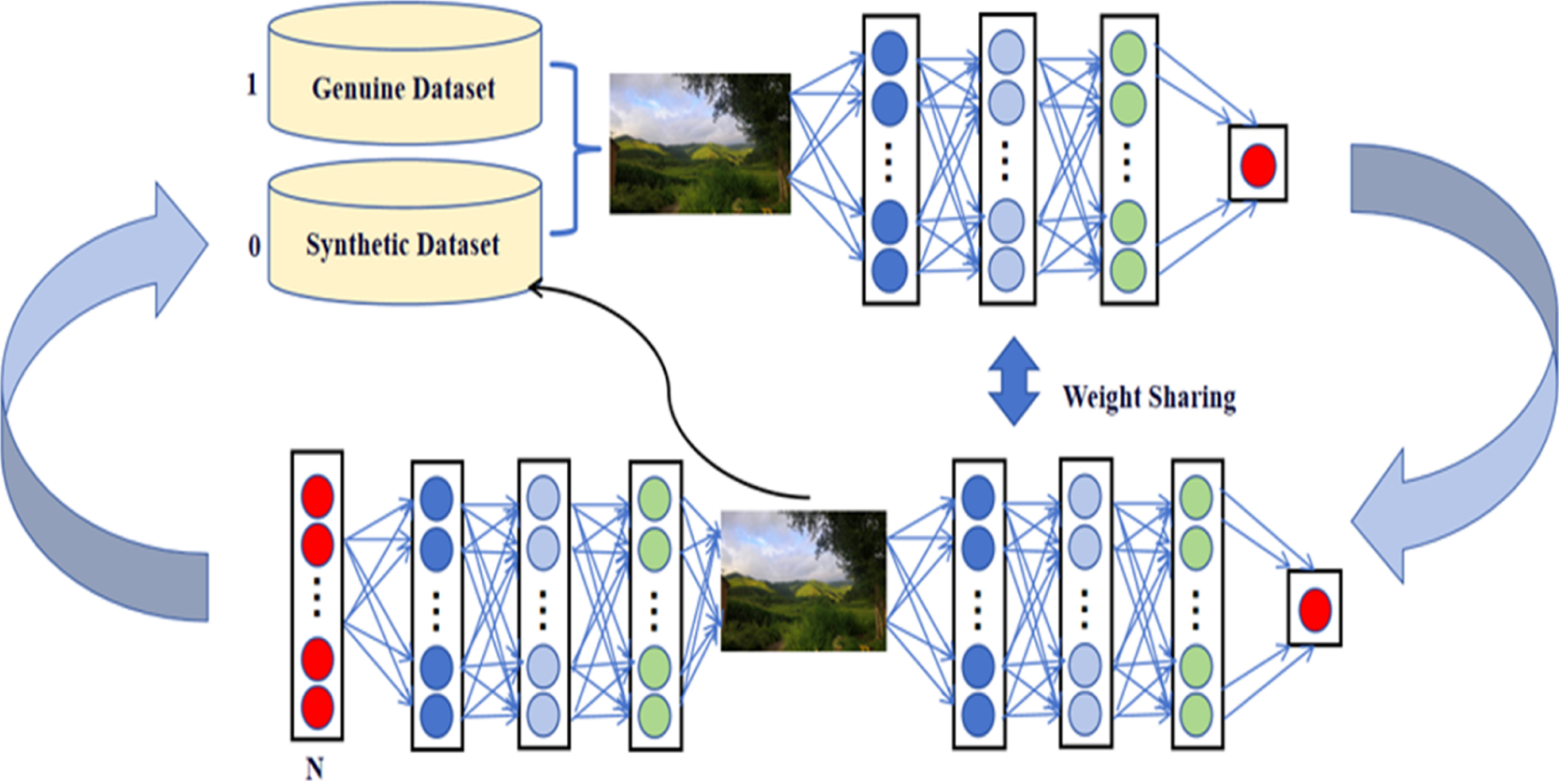
Fundamentals of GAN. Photograph courtesy of Yawei Ma. Copyright 2025.

**Figure 3:**
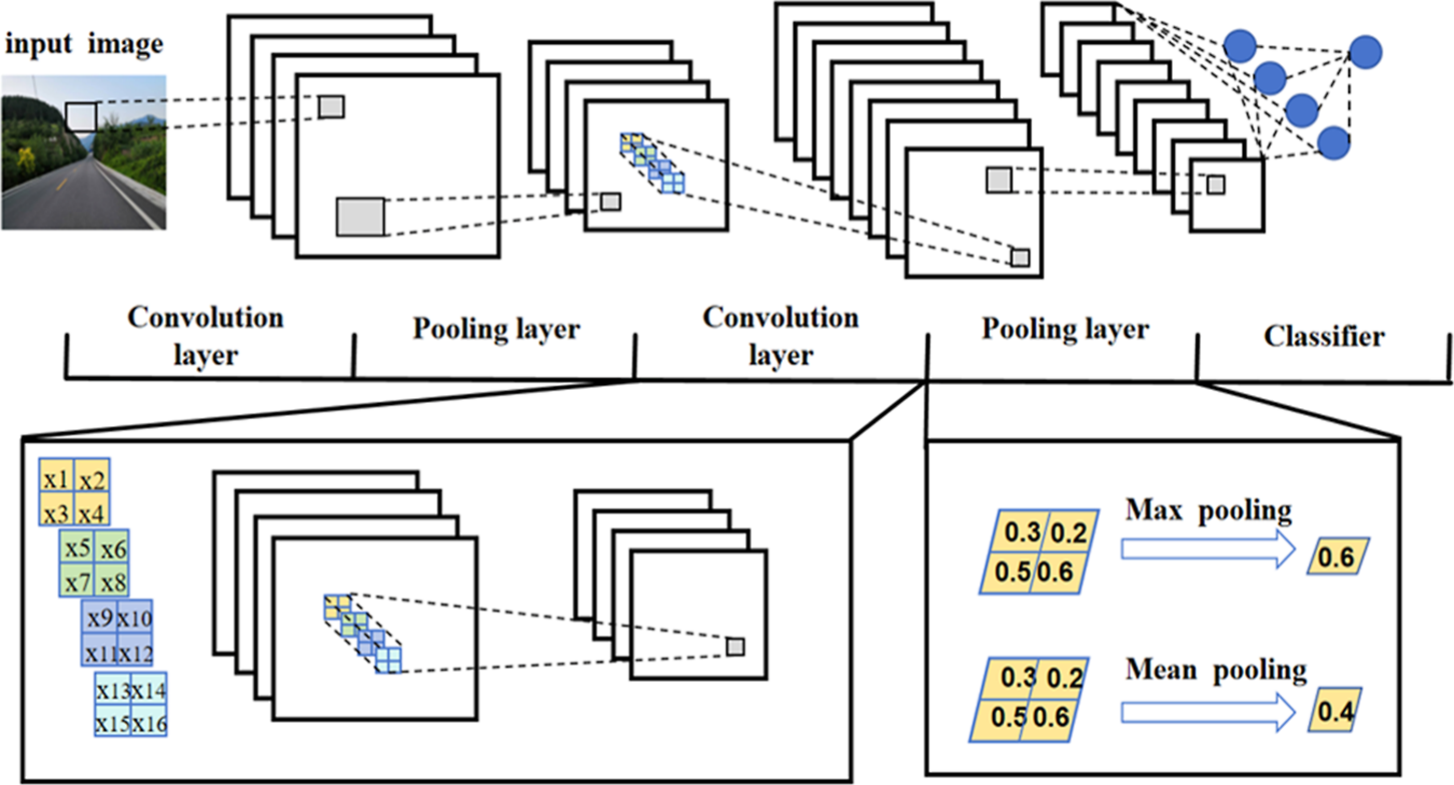
Schematic of a typical convolutional neural network (CNN), consisting of alternating convolution and pooling layers followed by a classifier. Convolution layers extract hierarchical features, while pooling layers (e.g., max or mean pooling) reduce spatial resolution and enhance invariance. The final feature maps are flattened and fed into a fully connected classifier, such as a multilayer perceptron. Photograph courtesy of Yawei Ma. Copyright 2025.

### 3.2 ML Tasks in Drug Discovery (Target Identification + Virtual Screening)

ML has emerged as a critical tool in early stage drug discovery, where it supports tasks ranging from target identification to VS. In target identification, ML models analyze high-dimensional omics data sets—including transcriptomics, proteomics, and interactomics—to detect disease-relevant molecular signatures or network perturbations. For instance, supervised learning models such as RF and SVM are used to classify potential drug targets^52^ based on gene expression^53^ or pathway activation features.

VS has revolutionized drug discovery by enabling the efficient computational screening of extensive compound libraries. It employs two primary methodologies: structure-based (structure-based virtual screening (SBVS)) and ligand-based (LBVS) approaches.^54^ ML can be integrated with LBVS or SBVS screening. While LBVS relies on similarity to known actives, ML enhances prediction accuracy through feature learning from chemical fingerprints or molecular descriptors. In SBVS, CNNs and graph-based models (e.g., graph convolutional networks (GCNs)) can learn directly from 2D/3D molecular structures or protein–ligand complexes.

SBVS evaluates ligand–target interactions through molecular docking, where compounds are computationally positioned at binding sites and scored using predefined metrics.^55^ LBVS, particularly valuable for targets like GPCRs, predicts binding potential based on molecular similarity principles using structural and physicochemical properties of known active/inactive molecules, necessitating unbiased benchmark test sets for validation.^56^ ML-driven VS has demonstrated remarkable success in rapidly identifying bioactive compounds from millions of candidates, optimizing existing molecules, and elucidating molecule–target interactions, thereby expanding therapeutic possibilities in drug development.

Recent advancements include quantum-enhanced SVMs, as demonstrated by Mensa et al.,^57^ who integrated classical SVM algorithms with quantum kernel estimation for applications including COVID-19­(Figure 4) drug discovery. Adeshina et al.^58^ developed vScreenML, an XGBoost-based SF classifier, which successfully identified potent AChE inhibitors (IC50 values down to 173 nM) without medicinal chemistry optimization. The effectiveness of ML-based SFs is further enhanced by large training data sets,^59^ with mega-compound libraries enabling the rapid discovery of selective, low-nanomolar potency drug precursors. ML methods have also been combined with docking, pharmacophore modeling, and quantitative structure–activity relationship (QSAR) techniques to prioritize the hit compounds. The choice of data representation—SMILES strings, fingerprints, or graphs—heavily influences model performance. Overall, ML facilitates a more data-driven, scalable, and cost-efficient approach to drug candidate generation, especially when integrated with high-throughput screening platforms.

**Figure 4:**
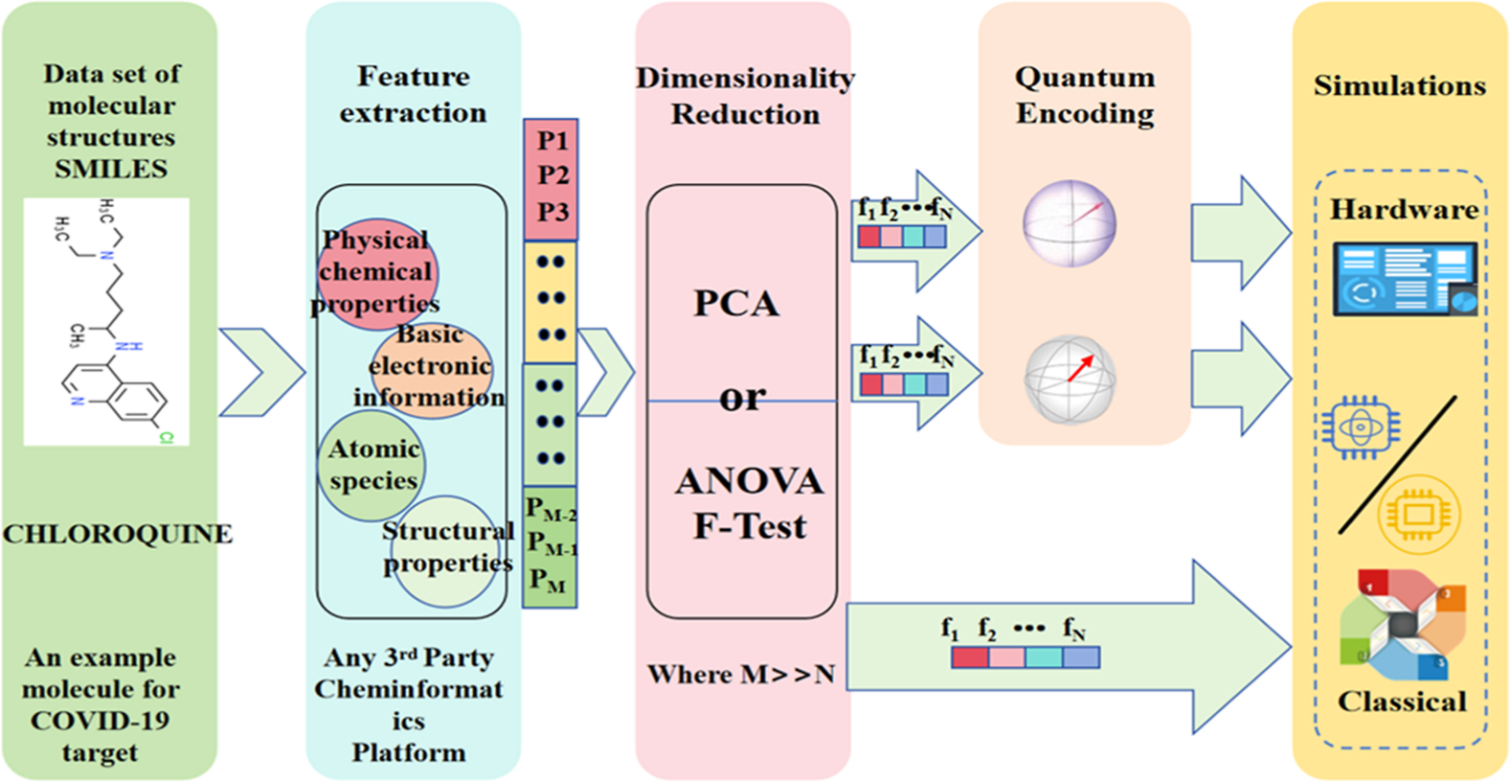
A hybrid classical-quantum integrated ML framework for ligand-based VS of drugs. A SMILES-encoded molecular database is utilized to extract various molecular features using RDKit. By refining the feature vectors, these are passed to the training and testing of support vector classifier algorithms, and this is named prospective quantum advantage in drug discovery.

### 3.3 ML for Drug Repositioning

Drug repositioning, also termed drug repurposing­(Figure 5), represents a strategic approach to identifying novel therapeutic applications for existing medications. This methodology leverages established pharmacological agents with well-characterized safety profiles, offering a time- and cost-efficient alternative to traditional drug development. The repositioning process typically integrates computational approaches (e.g., ML, network pharmacology, and bioinformatics) with experimental validation (in vitro and in vivo models), culminating in clinical trials to confirm the therapeutic efficacy. The established safety profiles of repurposed drugs significantly reduce development risks while accelerating the availability of novel treatment options.^60^ Several computational methods, including ML,^61^ network analysis^62^ and text mining,^63^ have been used for drug repurposing. ML-based repositioning strategies use a variety of data sources including gene expression profiles, chemical structures, protein interaction networks, and clinical annotations.

**Figure 5:**
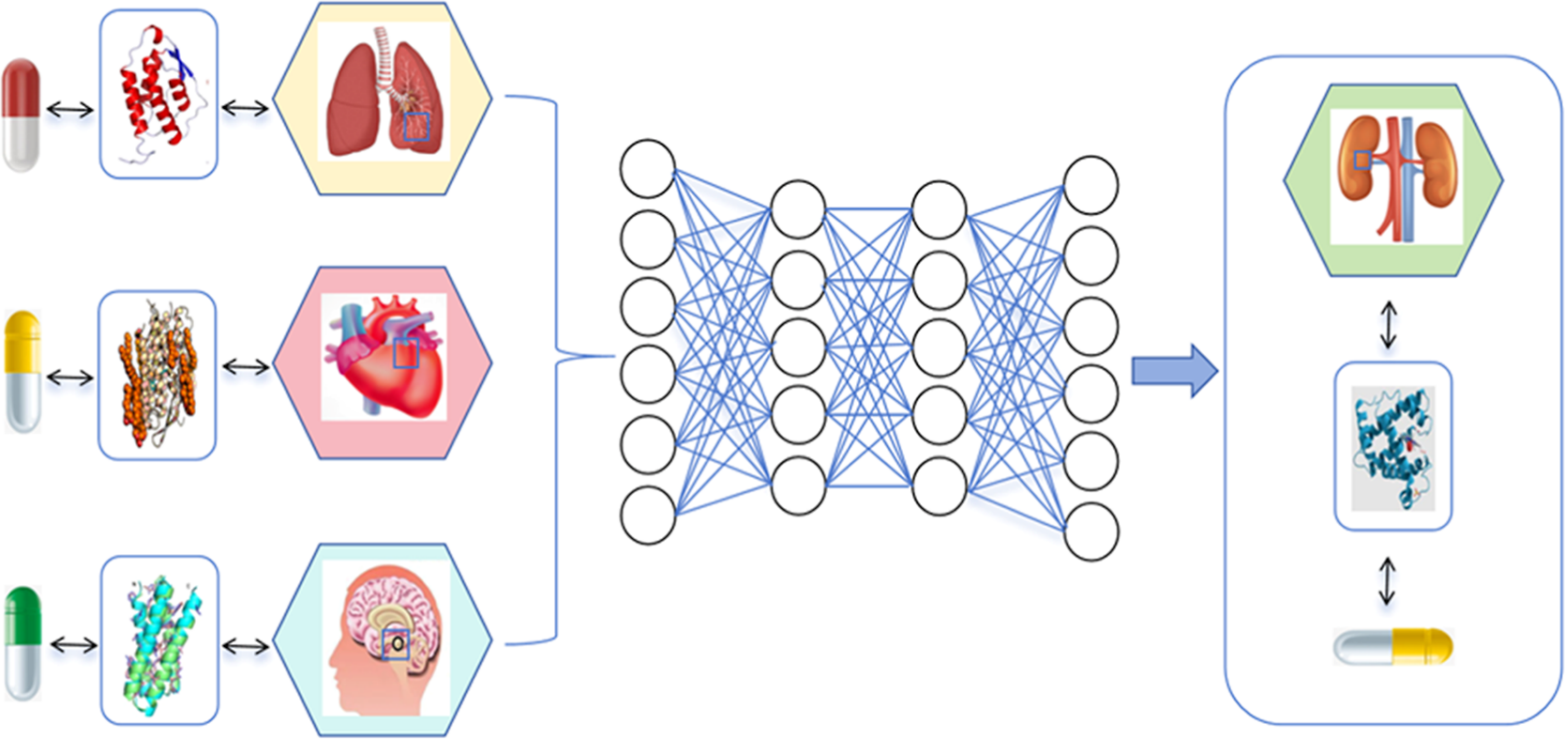
Drug repositioning using DL methods.

One common approach involves transcriptomic similarity matching, where ML models compare disease gene expression signatures with drug-induced signatures (e.g., from the Connectivity Map, CMap). Tools such as DeepCE and DRUG-seq integrate DL with transcriptome data to predict repositioning candidates. Another promising strategy is knowledge graph-based learning, where drugs, targets, diseases, and pathways are connected into heterogeneous networks. Graph neural networks (GNNs) and embedding methods like node2vec and DeepWalk are used to learn latent representations that support drug–disease association prediction. By combining biological similarity, topological proximity, and functional genomics, ML offers a powerful framework for systematically exploring new uses for old drugs.

Building on these graph-based frameworks, recent advances in high-throughput screening and large-scale omics data sets have further enabled the systematic exploration of drug repurposing opportunities in NDD contexts.^64^ Additionally, matrix factorization, autoencoders, and hybrid deep models have been applied to infer repositioning candidates from integrated omics and clinical data. These methods can prioritize drugs for experimental validation in diseases by identifying overlapping mechanisms, such as inflammation or oxidative stress.

### 3.4 ML in Drug Combination Prediction

Traditional monotherapies often face limitations due to side effects and drug resistance,^65^ prompting the development of combination therapies that can enhance efficacy while minimizing adverse effects. Drug interactions typically fall into three categories: antagonistic, additive, or synergistic, with synergistic combinations offering dose reduction and delayed resistance development.^66^ However, identifying effective drug combinations remains challenging due to the vast screening space. Recent advances in computational approaches have revolutionized drug combination discovery. ML models incorporating pharmacological and network topology features^67^ enable efficient screening of potential combinations from extensive drug libraries. ML algorithms can predict synergistic effects, optimize dosing regimens, and facilitate personalized treatment strategies based on individual patient profiles.

ML approaches for drug combination prediction can be broadly categorized into three main strategies:(1) feature concatenation models, such as DeepSynergy,^68^ which integrate compound descriptors (e.g., molecular fingerprints) with transcriptomic profiles of cell lines to predict synergy scores. Subsequent developments include DTF, which combines tensor factorization with deep neural networks, achieving comparable performance to DeepSynergy.^69^ SYNDEEP^70^ represents the state-of-the-art, utilizing comprehensive physicochemical and genomic data to achieve remarkable prediction accuracy (92.21%) and AUC (97.32%) in 10-fold cross-validation, outperforming traditional ML approaches (SNN, KNN, RF, SVM, GBC).(2) Interaction-aware architectures, exemplified by MatchMaker,^71^ which incorporate drug-target interactions and protein–protein interaction (PPI) networks to capture biological context. TranSynergy advanced the field by incorporating gene interaction networks and enabling mechanistic interpretation through CNNs.^72^ Zhao et al.^73^ propose an interpretable framework for DDI prediction by constructing a heterogeneous information network based on biological knowledge. A meta-path-based fusion mechanism is employed to learn drug representations, and an attention mechanism integrates semantic information from meta-paths of different lengths for accurate prediction. (3) Multitask ensemble models, including MARSY,^74^ which simultaneously learn from multiple objectives, such as classification, regression, and toxicity prediction, while integrating heterogeneous data modalities such as gene expression and pathway activity. These models predict synergy scores, facilitating the identification of multitarget therapies that simultaneously modulate multiple pathways, thereby reducing resistance risk and drug toxicity.^75^

ML has significantly transformed the landscape of neurodegenerative drug discovery, offering data-driven strategies to streamline early stage target identification, optimize compound screening, and identify novel therapeutic opportunities through repositioning and combination prediction. However, the success of ML-based approaches hinges not only on algorithm selection but also on rigorous evaluation, interpretability, and data quality. As research advances, the integration of multiomics data, explainable AI (XAI), and federated learning will likely enhance the robustness and clinical translatability of these models, paving the way for precision medicine in NDD therapy.

### 3.5 AI-Powered Target Identification

The development of therapeutics for NDDs, including Alzheimer’s, Parkinson’s, and Huntington’s diseases, focuses on identifying compounds that can halt or reverse neuronal degeneration. Identifying therapeutic targets remains a foundational step in drug discovery for NDDs. Recent research highlights promising targets such as striatal-enriched protein tyrosine phosphatase (STEP), PARP-1,^76^ MAO-B, GSK3β,^77^ and LRRK2,^78^ all of which are implicated in the pathogenesis of AD and PD. For instance, STEP is known to regulate *N*-methyl-d-aspartate (NMDA) receptor internalization,^79^ contributing to synaptic dysfunction in AD. Wang et al. constructed a diagnostic model^80^ for AD using RF algorithms and subsequently interpreted the model using SHapley Additive exPlanations (SHAP). The analysis revealed that MYH9 has emerged as a promising novel potential therapeutic target.

Elevated MAO-B activity in PD patients exacerbates dopaminergic system abnormalities by degrading monoamine neurotransmitters, including dopamine.^81^ Selegiline, a selective MAO-B inhibitor, has demonstrated efficacy as a PD adjunct therapy,^82^ highlighting MAO-B’s broader therapeutic potential in NDDs. Recent computational approaches have advanced MAO-B inhibitor discovery. ML models (SVM, NB, LR, RF) achieved 81% accuracy in identifying potential inhibitors, with lead-2 demonstrating neuroprotective effects in experimental and histological analyses.^83^ These findings underscore the value of integrating ML with traditional VS for target-specific lead compound identification.

Researchers constructed an AD-specific transcriptional profiling atlas and examined the gene expression responses of human-induced pluripotent stem cell (hiPSC)-derived cortical neurons treated with compounds selected from the CMAP cancer cell profiling data set.^84^ Advances in gene-editing technologies such as CRISPR-Cas9 and hiPSC models­(Figure 6) have facilitated high-fidelity disease modeling. When combined, these technologies allow for precise genetic manipulation and mechanistic validation in human-relevant systems.^85^ This integration of disease-relevant neuronal models with gene perturbation data sets facilitates the identification of promising therapeutic candidates for AD. In addition, Li et al. integrated DL-based GCNs^86^ with pharmacological analyses to elucidate the potential of pyrroloquinoline quinone as a neuroprotective agent for AD.

**Figure 6:**
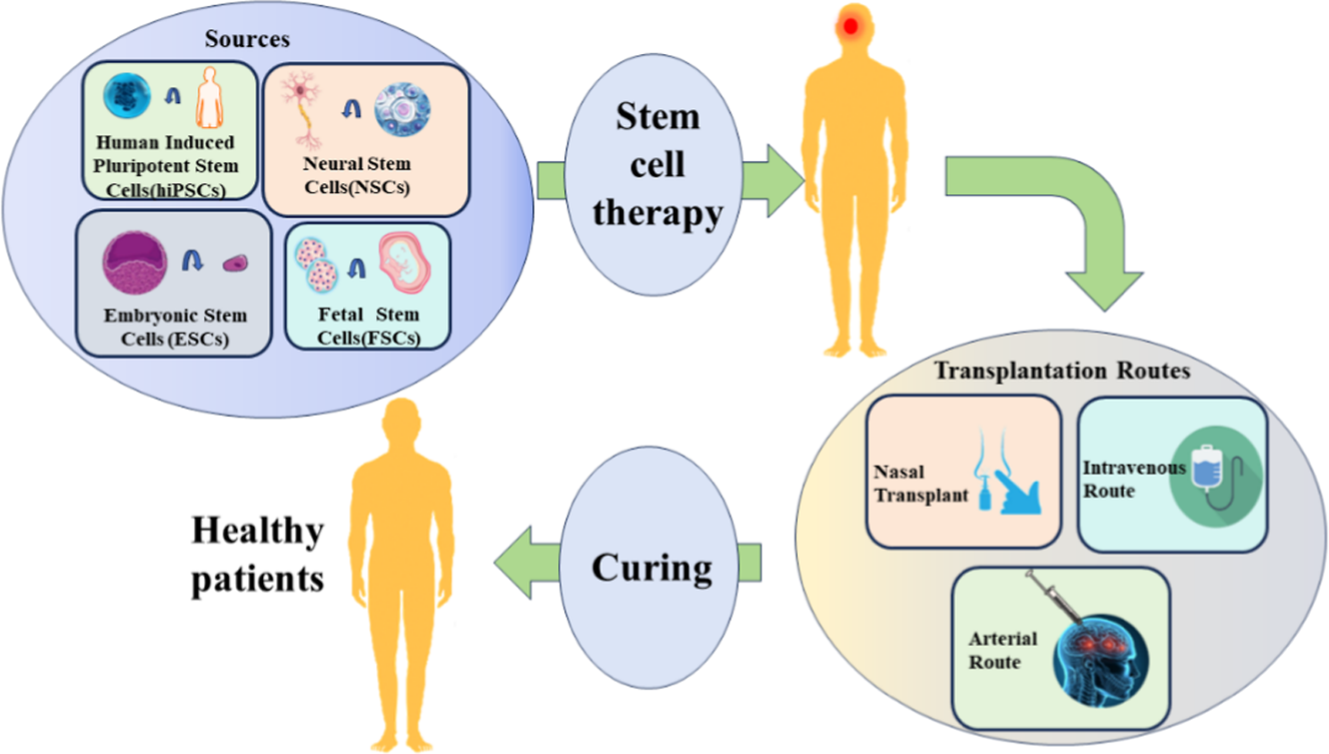
Stem cell therapy for patients with NDDs.

ML models like Cordax^87^ have shown utility in identifying aggregation-prone regions in proteins, while GCNs and heterogeneous network-based approaches such as HENA^88^ have enabled multiomics integration for target discovery. These tools enhance our capacity to elucidate disease mechanisms and prioritize biologically relevant targets.

## 4 Drug Discovery for NDDs: from Target to Candidate

NDDs pose formidable challenges to traditional drug discovery pipelines due to their multifactorial etiologies and complex clinical manifestations.^7,89^ The rapid integration of AI and ML into pharmaceutical research has significantly enhanced every stage of the discovery process—from target identification to lead optimization. This section explores key advances in AI-driven drug discovery for NDDs, emphasizing computational strategies that accelerate candidate selection and optimize therapeutic precision.

### 4.1 Virtual Screening and ML-Assisted Lead Discovery

Once potential targets are identified, the next step is the discovery of small molecules or peptides that modulate the target activity. SBVS and ligand-based approaches have been greatly accelerated by ML techniques. Notable examples include DNN and RF models for hit prediction, as demonstrated by Tsou et al.,^90^ who successfully screened out GPCR agonists and triple-negative breast cancer inhibitors from a large compound library and SVM applications in identifying dual-target ligands for A2A and D2 receptors.^91^ Hsieh et al.^92^ identified a PD therapeutic candidate through SBVS targeting the Miro1 protein, with one compound showing neuroprotective effects in preclinical models.

QSAR modeling using algorithms such as RF and SVM has been successfully applied to screen chemical libraries. Mukerjee et al. employed RF regression^93^ for QSAR analysis, combined with molecular dynamics simulations, and successfully identified stable Keap1 inhibitors with significant activity. Galati et al.^94^ developed ML models (KNN-Morgan, RF-Morgan) that identified GSK3β inhibitors with low micromolar to submicromolar activity. Similarly, casein kinase 1, implicated in Alzheimer’s pathology with 30-fold elevated levels in patient brains,^95^ was targeted through SBVS of 500,000 compounds, yielding three potent CK1δ inhibitors (ZINC48488295, ZINC04869366, ZINC4412706) with significant enzymatic suppression.^96^ Siddiqui et al. employed a light gradient boosting machine classifier to identify five candidate compounds capable of activating GPR40, thereby promoting hypothalamic cell proliferation and viability as a potential therapeutic strategy.^97^

In Alzheimer’s research, graph convolutional neural networks have identified BACE1 inhibitors,^98^ while deep neural networks have shown potential in classifying drugs based on transcriptional profiles. These approaches not only increase the efficiency of screening but also allow for mechanism-informed compound selection, while Aliper et al. employed D^99^ trained on transcriptional data to classify drug candidates for AD, emphasizing AI’s ability to stratify therapies based on cellular response profiles. Recent models also target specific molecular pathways. For example, Lee et al. developed a predictive model for S100A9 inhibition^100^ using two-dimensional molecular descriptors, and Cavas et al. used ANN to study acetylcholinesterase inhibition^101^ by carbazole derivatives, underscoring the adaptability of ML to diverse biochemical contexts.

### 4.2 toward Multi-Target and Precision Therapeutics

Given the multifactorial nature of NDDs, polypharmacology and precision medicine strategies are increasingly emphasized. Multitarget directed ligands are being developed to simultaneously target key pathological pathways, such as the combination of GSK-3β/DYRK1A^102^ or AChE/GSK-3β.^103^ These efforts reflect a shift from single-target to systems-level interventions.^104^ Alshabrmi et al. integrated ML and network pharmacology^105^ approaches to identify potential therapeutic compounds targeting two key proteins, MTOR and BCL2, for AD. Using RF and other models, the study screened candidate molecules from a library of 19,000 compounds. The selected candidates were further evaluated through molecular docking and molecular dynamics simulations, leading to the identification of 13 multitarget compounds with promising therapeutic potential. Recent ML models have also supported the development of peptide-based therapeutics, providing alternatives to traditional small molecules with improved selectivity and bioavailability. Platforms combining in silico prediction with wet-lab validation represent a paradigm shift in therapeutic development.

This section has outlined the evolving landscape of drug discovery for NDDs, driven by the integration of AI and ML techniques. From high-resolution target identification to structure-based compound screening and precision-guided therapy design, computational methods are accelerating the translation of basic research into viable clinical candidates. Future advances will continue to refine multitarget strategies, expand omics-based prediction, and integrate personalized data to meet the complex therapeutic demands of NDDs. Table 5 summarizes various ML/DL algorithms applied in neurodegenerative drug discovery, focusing on tasks such as diagnostic prediction, feature extraction, and VS. These approaches span different compound databases and validation strategies, showcasing the diversity and feasibility of AI in preclinical drug discovery.

**Table 5:** ML/DL Applications in Neurodegenerative Drug Discovery

ML/DL algorithm	task type	data set	evaluation metric	target/compound	experimental validation
RF	diagnostic	GEO(GSE109887)	test set: 0.95, validation set:0.79	MYH9	animal research^80^
GCN	feature extraction	original data set (11,542 compounds)	AUC-ROC:0.91	PQQ	animal research^86^
LGBMC classifier	hit screening	ZINC	AUC-ROC:0.92	compounds(1,3,4,6,10)	no^97^
SVM	hit screening	traditional Chinese medicinal (TCM) database	accuracy:0.81	lead-1 + lead-2	zebra fish model^83^
RF	hit screening	ChEMBL		DB06841	no^93^
KNN-Morgan/RF-Morgan	hit screening	ChEMBL30	accuracy: 0.71	G1+G4	in vitro^94^
GCNN	feature extraction + classification	binding DB	accuracy: 0.99	BACE1 inhibitors	in vitro^98^
ANN	regression model	original data set(351 compounds)		compounds(4,2a,2b)	in vitro^101^
RF	hit screening	binding DB	test set: 0.78, validation set:0.98	13 candidate compounds	no^105^
deep autoencoder/XGBoost	feature extraction + classification	DrugBank	AUC-ROC 0.661	187 AD targets/244 drugs	no^106^

## 5 Drug Repositioning and Target Network Modeling

Drug repositioning offers an efficient strategy for treating NDDs by repurposing existing drugs with known pharmacokinetic and pharmacodynamic profiles. Given the high failure rate of de novo drug development, the integration of AI and ML has become a powerful alternative. This chapter highlights recent computational advances in drug repurposing and network-based target discovery, emphasizing the role of large-scale data sets, multiomics integration, and graph learning in advancing therapies for AD, PD, and related disorders.

### 5.1 ML-Enabled Drug Repurposing for NDDs

Current pharmacological approaches for treating NDDs encompass symptomatic therapies and disease-modifying treatments. Notably, nonsteroidal anti-inflammatory drugs (NSAIDs), such as naproxen and ibuprofen, offer dual benefits by alleviating pain and attenuating neuroinflammation. Emerging evidence indicates that NSAIDs may reduce neuroinflammatory responses and the accumulation of senescent cells, thereby providing therapeutic potential beyond their conventional analgesic roles.^107^ Drug repurposing has emerged as a promising strategy in AD research, with several oncology and antimicrobial agents demonstrating notable neuroprotective effects. For instance, carmustine has been shown to significantly reduce amyloid-β production in APP-overexpressing cell models,^108^ while bexarotene effectively reversed neurodegeneration and lowered amyloid-β levels in transgenic AD mouse models.^109^ Similarly, tamibarotene, a retinoid receptor agonist, exhibited multitarget activities, including anti-inflammatory effects and behavioral improvements.^110^

In addition to these agents, paclitaxel, thalidomide, and various antimicrobial drugs have also shown therapeutic potential through distinct mechanisms, suggesting broad applicability of repositioned compounds in modulating AD pathology.^111^ Complementing these findings, ibudilast was found to confer neuroprotection via TLR4 and kinase pathways (Oliveros et al.),^112^ while sertraline, a widely used antidepressant, demonstrated multitarget inhibitory effects on AChE, COX-2, BACE-1, GSK-3β, and caspase-3 in AD models.^113^ ML has revolutionized drug repurposing by enabling large-scale analyses of gene expression profiles, electronic health records, and biomedical literature­(Figure 7). Techniques such as knowledge graph-based reasoning and AI-driven text mining facilitate the discovery of novel drug–target–disease relationships.

**Figure 7:**
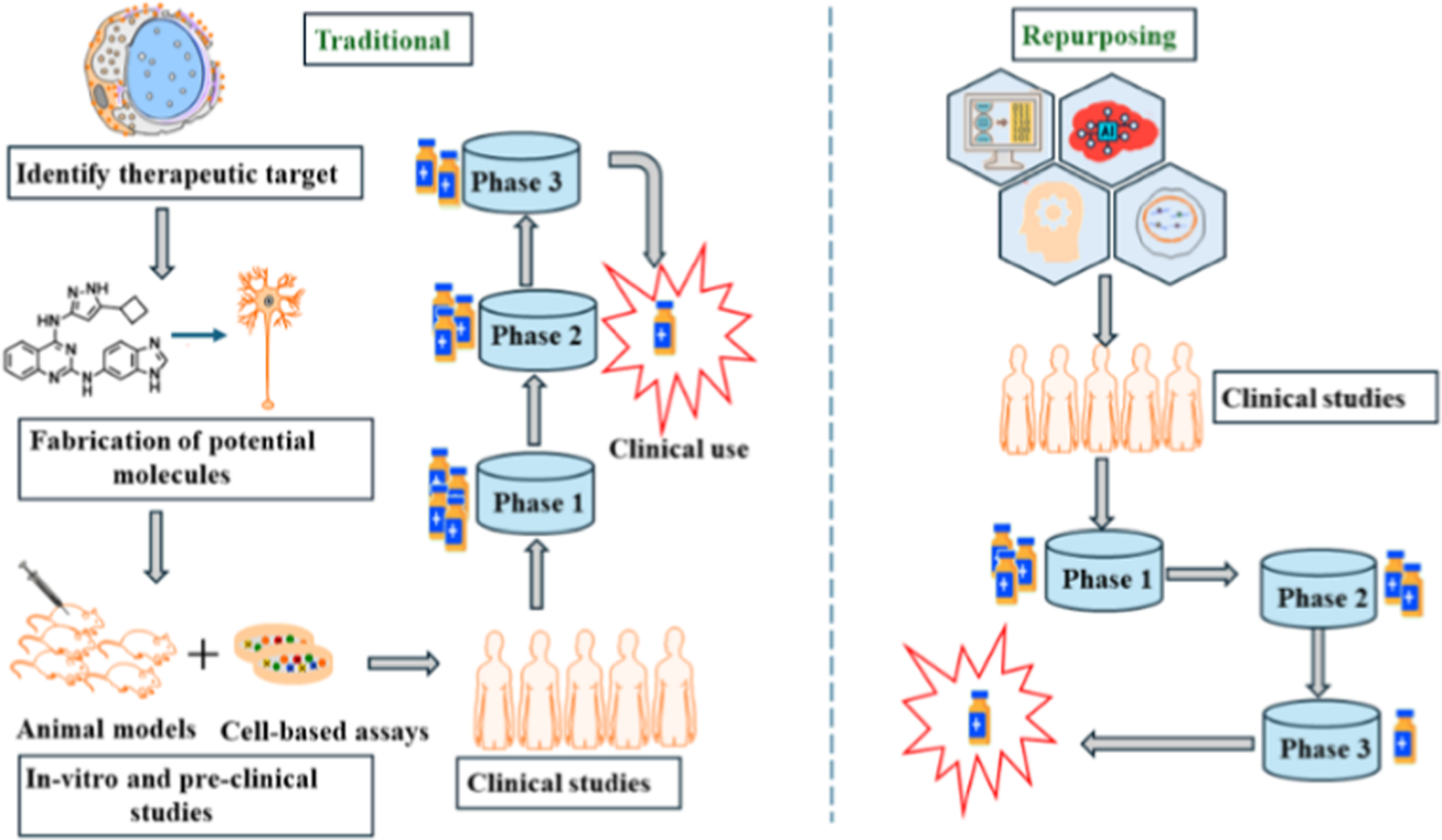
Drug processes reapplied with traditional and ML methods.

AlzhCPI,^114^ an ML-based web server employing recursive partitioning and Naïve Bayes algorithms, exemplifies predictive modeling for compound–target interactions in AD. Similarly, various studies have adopted SVM SVMs, RF, and DNNs to identify inhibitors targeting pathological enzymes and receptors such as acetylcholinesterase and S100A9.^115^ In the context of PD, Quan et al. proposed a network-based bidirectional drug repositioning approach based on PPI networks,^116^ identifying ten potential therapeutic candidates. Building on this network pharmacology strategy, Guo et al. identified 176 FDA-approved drugs with potential anti-Parkinsonian effects.^117^ Notably, two compounds, Omaveloxolone and cytoproheptadine, exhibited significant neuroprotective activity in both in vitro and in vivo models, primarily through the activation of the Keap1–Nrf2/ARE and MAPK/NFκB signaling pathways.

Advanced in vitro models, such as human midbrain organoids,^118^ combined with ML algorithms, have improved neurotoxicity screening and prediction of multitarget synergies. Recent progress in DL and GANs has further expanded the scope of repurposable chemical scaffolds with novel mechanisms.

### 5.2 Target Prioritization via Multi-Omics and Network Modeling

Target identification remains a major bottleneck in NDDs drug discovery. Orlenko et al. present an ML model integrated with biological network^119^ knowledge to identify novel gene-drug targets for AD. Leveraging multiancestry genomic data from the ADSP and SNP epistasis analysis, the model revealed significant interaction effects in noncoding region SNPs of CDC7 and CDC42, which are non-AD genes.

Fang et al. developed an integrated network-based AI strategy that synergizes GWAS data with multiomics profiling.^120^ This approach employs Bayesian inference and PPI network analysis to systematically prioritize high-confidence AD risk genes (ARGs) while evaluating their network proximity to established drug targets, leading to the identification of pioglitazone as a clinically actionable repositioning candidate. Building upon this framework, the NETTAG^121^ platform further advances the field through its interpretable DL architecture that comprehensively integrates multiomics signatures with human int-eractome mapping. This next-generation tool has successfully identified both novel AD-associated genes and repurposable therapeutics (notably gemfibrozil), with subsequent validation across transcriptomic/proteomic platforms and large-scale electronic health record data sets. Muslu et al. introduced GuiltyTargets,^122^ a positive-unlabeled learning method that integrates genome-wide PPI networks with disease-specific gene expression data. The model achieved AUC-ROC values of 0.92–0.97 across 12 data sets spanning six disease categories. In AD, GuiltyTargets identified catechol-*O*-methyltransferase (COMT) as a top candidate, aligning with its established role in PD therapy (e.g., tolcapone). This cross-disorder insight highlights the method’s translational relevance.

Tsuji et al. further advanced^106^ network-based prioritization by applying deep autoencoders and XGBoost classifiers to the Human PPI Network. Their model, augmented with SMOTE to mitigate class imbalance, identified several key targets (DLG4, EGFR, and RAC1) and repositionable compounds (tamoxifen, bosutinib). In a parallel study, Jacqueline et al. developed DOTA (Drug Repositioning via Optimal Transport for AD),^123^ which uses multimodal and Wasserstein variational autoencoders to evaluate the efficacy of circadian-active antipsychotics like quetiapine and aripiprazole. DOTA highlights a mechanistic link between circadian dysfunction and cognitive impairment in AD. MultiDCP,^124^ a DL model combining GCN (Figure 8), Transformer, and autoencoders, predicts drug-induced gene expression and dose–response curves. It identified mefenamic acid (anti-inflammatory) and neurotransmitter modulators as potential AD treatments, with experimental evidence supporting mefenamic acid’s neuroprotective effects in AD models. Rodriguez et al. introduced the DRIAD framework,^125^ leveraging logistic regression to identify 15 candidate drugs for AD, with partial in vitro validation. In contrast, Courtois et al. employed a large-scale ML analysis^126^ of the French national health database to detect marketed drugs—such as furosemide and smoking cessation agents—associated with reduced PD risk. Nitazoxanide^127^ has recently been demonstrated to exhibit therapeutic effects against PD.

**Figure 8:**
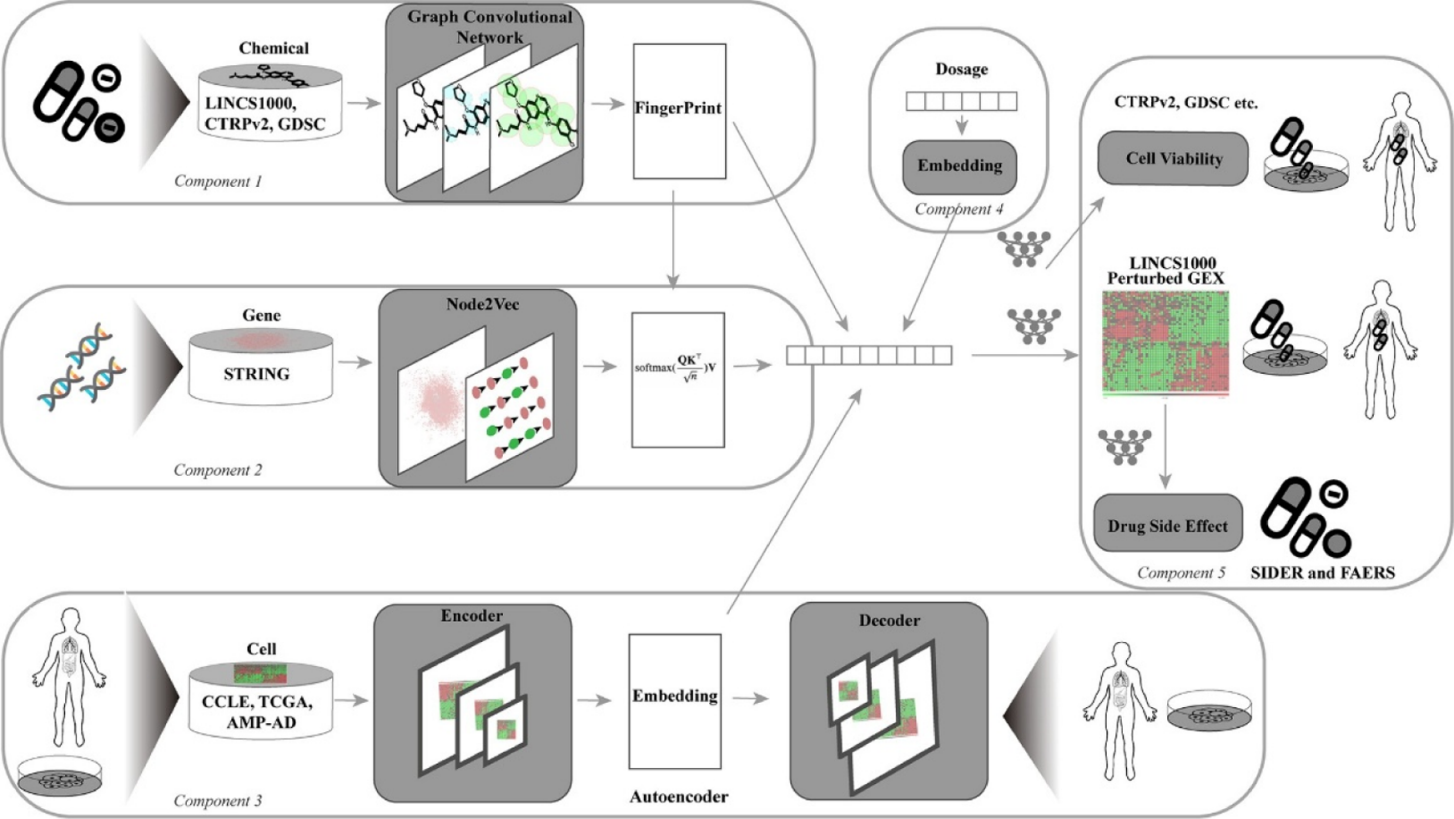
Architecture of MultiDCP and the cell line transformer. Reprinted from Wu et al.,^124^ PLoS Computational Biology, 2022, 18(8), e1010367. Copyright 2022 The Authors. Published by PLOS under CC BY 4.0.

Telmisartan, originally an antihypertensive agent, was successfully repurposed for AD through network-based classification approaches.^128^ In a similar vein, dolutegravir was identified via AI-driven screening as a promising candidate for AD treatment, highlighting the growing role of AI in systematic drug repurposing.^129^ Extending this paradigm, Temiz et al. combined network biology with ML to analyze transcriptomic data derived from ALS^130^ motor neurons and muscle tissues. Their integrative approach revealed two key gene clusters and predicted five repurposed drug candidates, including nilotinib and trovafloxacin, thereby providing novel therapeutic avenues for ALS.

AI and ML have enabled a paradigm shift in drug repositioning^131^ and target discovery for NDDs. From reanalyzing existing compounds to revealing novel targets through network and omics integration, computational frameworks offer scalable, cost-effective, and mechanistically informed pathways for therapeutic innovation. Future directions will likely emphasize real-world clinical validation, cross-disease generalization, and the fusion of multimodal biomedical data to support precision treatment strategies for AD, PD, and beyond. Table 6 presents examples of drug repurposing in NDDs, where approved drugs originally indicated for other conditions have been reassessed for AD, PD, or ALS. Table 4 highlights ML/DL-based strategies and their corresponding levels of experimental validation.

**Table 6:** ML/DL-Based Drug Repurposing for NDDs

original drug	original indication	new indication	database	ML/DL Algorithm	experimental validation
dolutegravir	antiretroviral agent	AD	SuperDRUG2	AI screening + molecular docking	in vitro^129^
ibudilast	antiasthmatic	AD	AMP-AD + LINCS L1000	3D-REMAP + Bayesian signature detection	in vitro kinase binding + APP/PS1 AD rat model^112^
sertraline	antidepressant drug	AD	ChEMBL	RF + GB + XGB	albino rat experiments^113^
gemfibrozil	lipid-lowering drug	AD	AlzGene	GNN + PPI	proteogenomics + 5xFAD rat model^121^
furosemide	variable selection + signal detection	PD	SNDS	subsampling + LASSO logistic regression	no^126^
nilotinib/trovafloxacin	chronic myeloid leukemia/bacterial infections	ALS	GEO	classification:SVM + MLP, regression: GB + SVR	phase 1 clinical trial^130^
mefenamic acid	anti-inflammatory	AD	LINCS L1000 + CCLE + TCGA + AMP-AD + DrugBank + STRING	GCN + feature extraction	preclinical research^124^
nitazoxanide	anti-infective drug	PD	ChEMBL + PubChem	XGBoost + Graph-CNN	no^127^

## 6 Combination Therapy: ML for Synergy Prediction

Drug combinations have become an important strategy in modern therapeutics, widely used in cancer treatment, and increasingly explored in NDDs. As precision medicine and personalized therapies advance, combination approaches are gaining renewed attention. In NDDs, combination therapy aims to enhance the treatment efficacy and minimize side effects by targeting multiple disease pathways. This section reviews current clinical evidence and highlights AI-driven methods for predicting synergistic drug combinations. In PD, combining zonisamide with standard antiparkinsonian drugs enhances symptom control.^132^ In ALS, the combination of ciprofloxacin and celecoxib has demonstrated synergistic effects in preclinical models.^133^ For AD, the widely used combination of donepezil (a cholinesterase inhibitor) and memantine (an NMDA receptor antagonist) effectively addresses both cholinergic deficits and glutamate excitotoxicity. Similarly, the efficacy of levodopa is enhanced when it is administered with dopamine decarboxylase inhibitors.

Recent advances in AI have transformed the prediction of synergistic drug combinations. Pan et al. introduced AI-DrugNet,^134^ a comprehensive framework­(Figure 9) integrating drug–drug, drug–target, and target–target relationships into a Drug–Target-Pathway network. Applied to a combinatorial library of over 2 million drug pairs, AI-DrugNet identified 233,269 potential AD drug combinations and highlighted five key target genes (AGTR1, FEN1, NADSYN1, PDHB, PSMB2) associated with brain injury and AD progression.

**Figure 9:**
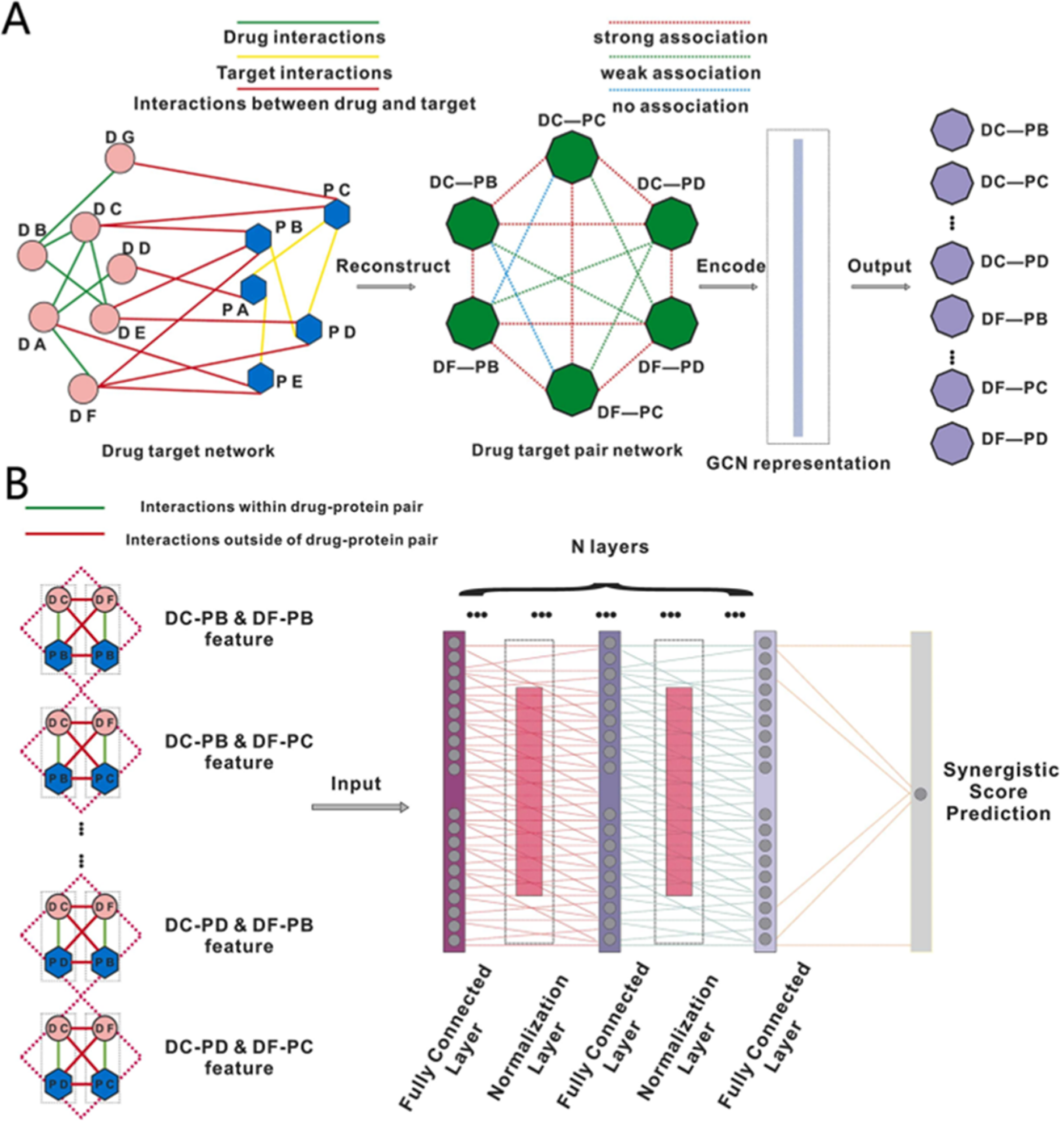
DL model for candidate drug combination discovery in AD. (A) Disease-specific drug-target pairs (DTP) network representation for DTP. (B) The architecture of the drug combinations prediction model. Reprinted with permission from Pan et al.,^134^ Computational and Structural Biotechnology Journal, 2023, 21, 1533–1542. Copyright 2023 Elsevier. Licensed under CC BY-NC-ND.

Building on this, Wang et al. developed DEML,^135^ a multitasking ensemble neural network that simultaneously addresses synergy regression, classification, and drug–drug interaction prediction. Its hybrid ensemble layer and task-specific fusion mechanism significantly outperformed prior models such as DeepSynergy, MatchMaker, and XGBoost in both RMSE and R2 metrics. Tang and Gottlieb proposed SynPathy,^136^ a pathway-informed DL model that improves both predictive accuracy and interpretability by leveraging biological pathway proximity between synergistic drug pairs. Similarly, Li et al. introduced DeepDrug,^137^ an AI-driven framework that integrates expert knowledge and biomedical graphs using GNNs. This approach identified a five-drug combination—tofacitinib, niraparib, baricitinib, empagliflozin, and doxercalciferol—targeting multiple pathological mechanisms of AD, offering a promising strategy for clinical translation.

AI-enabled synergy prediction models have greatly expanded the landscape of combination therapy development for NDDs. From empirical drug pairing to intelligent, mechanism-informed prediction, these models offer substantial promise for personalizing treatment regimens and optimizing therapeutic outcomes under complex neurodegenerative conditions. Table 7 outlines recent AI models developed for drug repositioning and drug combination prediction targeting of neurodegenerative disorders. These models leverage diverse algorithms and data sets to predict novel therapeutic associations, identify candidate drugs, and estimate synergistic effects.

**Table 7:** AI Models for Drug Repositioning and Combination Prediction

model	ML/DL algorithm	use case	performance	database	input	output
NETTAG^121^	GNN	drug repositioning	AUC:0.81	AlzGene	multiomics features + PPI network integration	predicted AD-risk genes + gene Scores
GuiltyTargets^122^	Gat2Vec + PU learning	drug repositioning	AUC:0.92–0.97	STRING(v10.5) + HIPPIE (v2.0)+GEO + AMP-AD	feature extraction + drug targets	likelihood score + ranked list of targets
DOTA^123^	multimodal autoencoder (MAE) + wasserstein variational autoencoder (WAE)	drug repositioning	test set: 0.85, validation set:0.95	DrugBank + therapeutic target database + PharmGKB	drug network data	drug-AD association scores + drug candidate
MultiDCP^124^	GCN + PU learning	drug repositioning	ROC-AUC:0.697	LINCS L1000 + CCLE + TCGA + AMP-AD + DrugBank + STRING	gene expression data + drug data	predicted expression
DRIAD^125^	LR	drug repositioning	AUC-ROC: 0.6–0.8	AMP-AD	differentially expressed gene list(DGL)	drug candidate list
AI-DrugNet^134^	GCN	drug combination	AUC-ROC: 0.98 ± 0.025	DrugBank + HIPPIE	feature extraction	drug-AD association scores
DEML^135^	DNN	drug combination	AUC-ROC:0.89	DrugComb+ Iorio research + DrugBank	chemical descriptors + cell line gene expression profiles	regression task + classification task
SynPathy^136^	DNN	drug combination		DrugComb + STRING + GDSC (cell line genomics)	chemical structure + PPI network + gene expression	predicted low synergy score
DeepDrug^137^	GNN	drug combination	AUC:0.977	DrugBank + STRING + DrugCentral + ChEMBL + BindingDB	heterogeneous bio-graph	drug-gene scores + drug combination scores

## 7 Explainable AI (XAI) in Neurodegenerative Disease Research

In recent years, explainable AI (XAI) has attracted growing attention in biomedical ML, offering substantial potential to improve our understanding of NDDs and to facilitate more effective treatment strategies, ultimately enhancing patient quality of life and therapeutic outcomes.^138^ By integrating diverse data modalities—including imaging, genomics, clinical, and demographic information—XAI enables the construction of comprehensive models that can predict how NDDs progress and how patients may respond to treatment. This allows researchers and clinicians to better understand how complex models generate predictions, thereby increasing trust and guiding subsequent biological validation. Moreover, Viswan et al. reviewed studies over the past decade that applied XAI to AD diagnosis,^139^ emphasizing the wide adoption of XAI for this purpose. They also presented several ML and DL models that incorporate XAI frameworks to enhance the interpretability and transparency of AI-based predictions.

Several categories of XAI techniques are particularly relevant (i) feature attribution methods (e.g., SHAP, LIME) that quantify the contribution of individual variables (ii) gradient- and attention-based methods (e.g., saliency maps, Grad-CAM, Integrated Gradients, attention mechanisms) that highlight important regions in imaging or sequential data; and (iii) rule- and causality-based frameworks (e.g., decision trees, counterfactuals, causal inference combined with ML) that provide human-readable or causal explanations. Table 8 summarizes representative XAI methods, their principles, and their applications in NDDs research.

**Table 8:** Representative Explainable AI (XAI) Methods and Applications in NDDs Research

method	principle	strengths/limitations	example applications in NDDs
SHAP	game-theoretic feature attribution	global + local interpretability; high computational cost	identify top clinical/omics features driving AD or PD predictions
LIME	local surrogate linear models	intuitive case-levelexplanations; unstable for some samples	explain individual MRI or patient-level risk predictions
grad-CAM/saliency	gradient-based visualization of model focus	strong for imaging; sensitive to noise	highlight the hippocampus or substantia nigra in AD/PD MRI
integrated gradients	path-integrated gradients between input and baseline	theoretically sound; baseline choice critical	quantify multimodal (imaging + clinical) feature contributions
decision trees/causal ML	rule-basedor causal inference combined with ML	human-readable; limited accuracy/strong assumptions	generate interpretable rules (e.g., APOE4 carriers at high AD risk)

Mahmud et al.^140^ demonstrated that transfer-learning ensembles (VGG16 + VGG19, DenseNet169 + DenseNet201) not only achieved high diagnostic accuracy for AD but also leveraged Grad-CAM and saliency maps to highlight image regions of pathological relevance. Similarly, Rahim et al.^141^ fused longitudinal dynamic images with cognitive attributes using a CNN–Bi–LSTM hybrid, reporting improved AUC values and using visual XAI explanations to reveal which temporal windows and frames contributed most to AD progression prediction. Almohimeed et al.^142^ extended this direction to cognitive subscores, showing that multilevel stacking can reach ∼92% accuracy while XAI explanations pinpoint the most informative subscores. Kamal et al.^143^ moved beyond imaging alone by combining MRI-CNNs with microarray classifiers, where LIME was used to interpret gene-level contributions, linking molecular signatures to imaging phenotypes.

In parallel, Bhandari et al.^144^ focused on PD diagnosis from gene-expression data, applying the least absolute shrinkage and selection operator (LASSO) with LR/SVM classifiers and SHAP to uncover key biomarkers, while Pianpanit et al.^145^ systematically compared interpretation strategies for SPECT-based PD models, highlighting guided backpropagation and SHAP as the most consistent. For ALS, Founta et al.^146^ combined causality-aware SES with XGBoost/RF and SHAP to classify ALS subtypes and interpret underlying molecular differences, whereas Müller et al.^147^ integrated SHAP into deep models of ALS progression, allowing prognostic drivers such as respiratory decline to be interpreted at the patient level. Antoniadi et al.^148^ further demonstrated clinical translation by embedding SHAP explanations into a decision-support system (C-ALS), which not only predicted quality-of-life risk but also provided human-readable guidance to clinicians. Table 9 summarizes representative recent studies that applied explainable AI (XAI) to NDDs modeling across multiple data modalities.

**Table 9:** Representative Recent Studies Applying XAI in NDD Modeling

study (year)	data/modality	model/strategy	XAI method(s)	data set	key results/notes
**Mahmud**et al.**, 2024**	MRI/image-based transfer learning (image data sets implied)	deep transfer-learning ensembles: ensemble-1 (VGG16 + VGG19), ensemble-2 (DenseNet169 + DenseNet201); top single model reported	saliency maps; grad-CAM	kaggle	ensembles ≈95% (accuracy/precision/recall/F1); best single model ≈96% accuracy. XAI (Grad-CAM/saliency) used to visualize image regions supporting AD predictions
**Rahim**et al.**, 2023**	ADNI multimodal: longitudinal dynamic 2D images (3 time steps) + cognitive attributes; *n* = 1692	hybrid CNN–Bi-LSTM fusion; 10-fold CV	visual XAI (saliency/heatmaps)	ADNI	AUC-ROC ≈94%; multimodal fusion improved AUC by ∼ 2%. XAI provided visual explanations for image–temporal contributions
**Almohimeed**et al.**, 2023**	ADNI cognitive subscores (neuropsychological subscales)	multilevel stacking on selected feature subsets	posthoc XAI explanations reported (feature-level)	ANDI	two-class (AD vs CN): accuracy ≈92.08%; three-class (AD/CN/stable-MCI): ≈90.03%. XAI used to interpret which subscores drive decisions
**Kamal**et al.**, 2021**	MRI images + microarray gene expression	MRI: SpinalNet and CNN; gene: KNN/SVC/XGBoost; multimodal comparison	LIME (gene-level explanations)	kaggle+ OASIS + NCBI	CNN accuracy reported 97.6% (higher than SpinalNet); SVC best for gene data; LIME highlighted gene predictors important for AD classification
**Bhandari**et al.**, 2023**	aggregated gene-expression data sets (PD)	LASSO feature selection + LR/SVM classifiers	SHAP (global, model-agnostic)	NCBI (GEO)	Best diagnostic performance with LASSO + LR/SVM; SHAP identified key biomarkers and provided global interpretability for SVM
**Pianpanit**et al.**, 2021**	SPECT images for PD recognition	several deep CNN architectures evaluated	compared six interpretation methods (incl. Guided backpropagation, SHAP)	public SPECT image data set + PPMI	Evaluation showed guided backpropagation and SHAP are appropriate in different contexts for SPECT-PD models
**Founta**et al.**, 2023**	gene expression (ALS and subtypes)	causality-aware feature selection (SES)→ XGBoost/RF	SHAP for classifier explanation	two third-party data sets	SES reduced gene set; SHAP explained classifier decisions and highlighted subtype-relevant genes; framework enables interpretable ALS classification
**Müller**et al.**, 2021**	longitudinal clinical data (ALS progression)	multiple DNNs for progression modeling	SHAP to explain predictions	Portuguese ALS patients	Combining DL with SHAP yielded accurate prognostic models and interpretable drivers of respiratory decline
**Antoniadi**et al.**, 2022**	clinical data (ALS/MND QoL prediction)	clinical decision support system (C-ALS) prototype	SHAP for local (per-case) explanations	local data set	SHAP provided posthoc local explanations with verbal guidance; enhanced clinician interpretability of QoL risk predictions

By integration of imaging, genomic, and clinical data, XAI improves diagnostic performance while enhancing model transparency. Key techniques, such as feature attribution, gradient- and attention-based approaches, and causality-driven frameworks, allow researchers and clinicians to interpret model decisions and identify biologically meaningful patterns. Applications in AD, PD, and ALS demonstrate XAI’s potential to reveal biomarkers, highlight pathological regions, and support prognostic evaluation. Importantly, XAI provides human-readable explanations that foster clinical trust and facilitate translation into patient care. Overall, XAI is emerging as a critical tool for bridging complex computational models with clinical decision-making in NDDs.

## 8 Diagnostics and Non-Pharmacological Innovations

As the clinical landscape of NDDs continues to evolve, there is a growing need for noninvasive diagnostic tools and alternative therapeutic strategies. This section reviews technological advances in diagnostics and explores promising nonpharmacological treatments including natural products and photobiomodulation (PBM).

### 8.1 Diagnostic Advancements in NDDs

Noninvasive and scalable diagnostic methods are crucial for the early detection and monitoring of NDDs. Cognitive and structural MRI modalities have become cornerstones of dementia research, offering high-resolution insights into brain atrophy and functional connectivity.^149^ The Addenbrooke’s cognitive examination has demonstrated superior sensitivity for AD and mild cognitive impairment (MCI) when compared to MoCA and MMSE at optimal thresholds.^150^ The Frontal Assessment Battery further enables evaluation of executive function, which is closely tied to frontal lobe integrity. Emerging tools include EEG-based biomarkers supported by wearable systems and mobile cognitive testing applications that aid in differential diagnosis among AD, MCI, and other dementias.^151^ Advances in genomics, powered by AI and DL algorithms, are enhancing variant effect prediction, stratifying patients for clinical trials, and improving prognostic precision.

An edited volume,^152^ Recent Advances in the Treatment of Neurodegenerative Disorders, synthesizes expert perspectives on pharmacological and complementary therapies—including Ayurveda and phytochemicals, vitamin therapy for PD, medicinal mushrooms, MS and ALS management, and nanoparticle/nanoformulation strategies. These comprehensive reviews place individual natural-product discoveries and formulation advances into a wider translational context, linking computational prioritization with preclinical and clinical development efforts.

Natural products, especially those derived from traditional Chinese medicine (traditional Chinese medicinal), have attracted attention as neuroprotective agents. Xie et al. employed AI-based molecular screening to identify 18 candidate AD therapeutics from 3724 natural compounds.^153^ Xiao et al. used network pharmacology and molecular docking to elucidate the mechanistic basis of traditional formulations.^154^ Researchers employed^155^ SVM–recursive feature elimination (SVM-RFE) and LASSO regression models to identify key genetic biomarkers. Additionally, the Integrative Traditional Chinese Medicine database was referenced to explore traditional Chinese medicines associated with lipid metabolism and their potential links to AD. This integrative approach led to the identification of four potential biomarkers: choline O-acetyltransferase, RAS oncogene family member (RAB4A), acyl-CoA binding domain-containing protein 6 (ACBD6), and alpha-galactosidase A (galactosidase A).

Recent preclinical studies highlight the autophagy-modulating and neuroprotective potential of TCM-derived extracts. In a rotenone-induced Parkinsonian mouse model, Rai et al. showed that oral *Mucuna pruriens* seed extract (l-DOPA ≈6.5% w/w) improved motor performance, reduced oxidative stress, and modulated key pathways by lowering mTORC1, restoring TFEB, enhancing Gcase activity, and increasing TH levels.^156^ These findings suggest that *M. pruriens* is a promising candidate for neuroprotection, although active constituents remain to be defined, and clinical validation is still needed.

PBM, first studied in 1967, has shown capacity to modulate neuronal and microglial activity.^157^ Animal models demonstrate delayed neurodegeneration and enhanced mitochondrial function following PBM exposure. Recent clinical efforts, receiving transcranial photobiomodulation^158^ treatment, have been shown to enhance cognitive function and brain-derived neurotrophic factor levels in adults with MCI, offering a noninvasive adjunct to conventional therapies. These alternative modalities underscore the potential of integrative strategies in managing neurodegeneration, particularly when they are supported by AI-enhanced screening and validation techniques.

### 8.2 Relevant Data Sources, Databases, and Software for Drug Discovery and Applications

Public biomedical data sets are essential for ML-driven drug discovery. Preprocessing and standardization of these data sets ensure model reliability and reproducibility. Numerous curated and open-access repositories provide multiomics, chemical, pharmacological, and neuroimaging data essential for training and validating ML models. Table 10 summarizes key publicly available databases relevant to drug screening and NDDs research. These databases provide a robust foundation for the development of predictive models in precision medicine. Their integration enables multimodal ML pipelines combining omics, pharmacological, and clinical imaging features to accelerate drug discovery and optimize therapeutic interventions, particularly in oncology and NDDs research.

**Table 10:** Categorized Public Databases for ML-Based Drug Discovery and NDD Research

category	database	data type	key content	main applications
**genomics & transcriptomics**	GEO	microarray, RNA-seq	global gene expression data from various organisms	biomarker discovery, expression analysis
	TCGA	multiomics (mRNA, miRNA, methylation, mutations)	cancer genomics for 33 tumor types	target identification, prognosis modeling
	AMP-AD	multiomics (AD-specific)	proteomic, transcriptomic, genomic profiles in AD brain tissue	mechanism analysis, target discovery
	ROS/MAP	longitudinal clinical and genomics	cognitive data + brain tissue omics	aging, dementia progression research
	PPMI	genomics, imaging, biofluids	longitudinal PD progression markers	PD modeling, early prediction
	Answer ALS	ALS multiomics	iPSC-derived multitissue data sets	ALS subtype classification
**chemical & drug information**	ChEMBL	bioactivity, targets	drug-like molecules, assay data, targets	QSAR, screening models
	PubChem	chemical structures, bioassays	>100 M small molecules	fingerprint extraction, similarity search
	ZINC	ready-to-screen compound structures	2D/3D purchasable compounds	virtual screening, docking
	DrugBank	chemical + pharmacological	detailed info on drugs and targets	drug annotation, PK/PD modeling
**drug combinations**	DCDB	approved & experimental combinations	mechanism and synergy data	drug synergy prediction
	FDA orange book	approved drug products	fixed-dose drug combinations	clinical validation
	NCI-ALMANAC	anticancer combos	in vitro dose–response matrices	oncology combo modeling
	ONEIL	oncology dose–response	>500 drug pairs, synergy scores	deep learning training data
	DREAM-AZ	challenge-based oncology combos	community-submitted predictions	model benchmarking
**neurodegenerative data sets**	ADNI	imaging, biomarkers, clinical	MRI, PET, CSF, genotypes for AD	early AD detection, progression modeling
	ADDI	AD data integration	global platform aggregating AD data	federated learning, multisite ML
	ADNP	longitudinal data	imaging + molecular AD markers	long-term tracking studies
	OASIS	open-access MRI	aging and dementia populations	structural biomarker research
	HABS	MRI, cognition	harvard-based aging brain study	aging-related neurodegeneration
	DementiaBank	language, speech	transcripts from dementia patients	NLP-based screening, classification
	mayo clinic (MCSA)	cognitive aging + biospecimens	cognitive trajectories in aging	cognitive decline prediction

NDD diagnostics and therapies are entering a new era shaped by digital technologies and precision approaches. From MRI and EEG to genomics and phototherapy, nonpharmacological innovations are providing critical support to traditional drug-based interventions. Continued integration of AI will likely drive the development of more targeted, early stage interventions, ultimately enhancing patient outcomes in complex neurological disorders.

## 9 Conclusions

### 9.1 Challenges and Limitations

Despite the progress made in applying computational methods to NDDs research, significant challenges remain. Traditional animal and cellular models often fail to capture the full complexity of diseases, such as AD, PD, and ALS, resulting in poor translation to clinical applications. ML models are similarly constrained by the limitations of available data sets, which are often small, inconsistently annotated, and lacking in multimodal integration. Another critical issue involves ethical considerations and data privacy in clinical neurology. The integration of AI into healthcare requires the use of sensitive patient information such as imaging data, genomic profiles, and electronic health records. These data are often heterogeneous and have limited representativeness, which can induce model bias and reduce generalizability. Even after deidentification, high-dimensional biomedical data remain susceptible to reidentification, and data breaches or model theft pose concrete security threats. Safeguarding these data against misuse, leakage, or unauthorized access therefore remains an urgent unresolved challenge, while transparent regulatory and oversight frameworks are still being refined. Moreover, research gaps remain in translating ML models into real-world clinical practice. Most algorithms are validated retrospectively, with few prospective trials demonstrating their clinical utility.

DL models, though powerful, frequently suffer from overfitting and limited generalizability due to small sample sizes and variability in imaging protocols. The privacy-sensitive nature of neuroimaging and genomic data further restricts open access, limiting opportunities for robust model training. Additionally, the “black box” nature of many ML models—particularly deep neural networks—raises critical concerns about interpretability and clinical trust. Data bias, noisy inputs, and algorithm robustness also present major obstacles, especially in real-world deployment scenarios. To address these issues, methods such as transfer learning, unsupervised feature extraction, and GANs are being explored for data augmentation and domain adaptation. Moreover, a lack of standardized evaluation metrics and reliance on single-data set validation impede model comparability and generalizability across studies. A more robust framework incorporating multiple independent test sets is essential to accurately assess model performance. Finally, the regulatory landscape presents another layer of complexity. The use of AI-driven tools in medicine demands rigorous evaluation of safety, fairness, and accountability. However, current regulatory guidelines are not yet fully adapted to rapidly evolving ML applications, resulting in uncertainty for both researchers and clinicians. Addressing these multifaceted challenges is crucial to ensuring that ML can be responsibly and effectively integrated into the diagnosis and treatment of NDDs.

To mitigate these risks, a combination of technical and governance measures is recommended: improve informed consent procedures (e.g., broad or dynamic consent); implement strict pseudonymization and data-minimization practices; employ controlled-access mechanisms or secure data enclaves; where appropriate, explore privacy-preserving techniques and explicitly report their trade-offs; adopt explainability tools and transparent reporting (e.g., model cards/datasheets) to clarify intended use and limitations; and prioritize multicenter external validation, prospective evaluation, and continuous postdeployment monitoring. Finally, establishing data access committees, defining clear accountability frameworks, and involving clinicians and patient representatives early in the research process are essential steps to ensure that AI tools are safe, equitable, and suitable for clinical practice.

### 9.2 Outlook

Looking forward, the application of ML in both diagnostics and drug discovery for NDDs holds great promise. Recent trends indicate a shift toward integrated approaches that combine multiple algorithms—such as ensemble methods—with optimization and feature selection techniques. These strategies improve diagnostic robustness and facilitate the extraction of clinically meaningful insights from high-dimensional data sets.

In clinical contexts, ML can enhance personalized medicine through predictive modeling of treatment outcomes and intelligent therapeutic planning. This includes the development of individualized treatment regimens based on genetic, imaging, and lifestyle data. Meanwhile, ML-supported frameworks for drug repurposing and multiomics integration are accelerating therapeutic discovery pipelines. To realize this potential, continued interdisciplinary collaboration among computational scientists, clinicians, and regulatory bodies is crucial. Equally important are investments in data quality, standardization, and interpretability-focused model design. The future of NDD research will likely involve a paradigm shift from isolated experimental approaches to AI-driven strategies that integrate biological, clinical, and computational intelligence. This shift could fundamentally reshape how we understand, diagnose, and treat NDDs.^159^

## List of Abbreviations

NDDs: neurodegenerative diseases
PD: Parkinson’s disease
MSA: multiple system atrophy
DLB: dementia with lewy bodies
AI: artificial intelligence
CMap: connectivity map
DRUG-seq: drug repurposing using gene expression signatures
VAEs: variational autoencoders
SVM: support vector machines
RF: random forests
XGBoost: eXtreme gradient boosting
KNN: K-nearest neighbors
ANN: artificial neural networks
CNN: convolutional neural networks
DBN: deep belief networks
SBVS: structure-based virtual screening
QSAR: quantitative structure–activity relationship
DRIAD: drug repurposing in Alzheimer’s disease
MultiDCP: multitask deep learning for compound profiling
t-PBM: transcranial photobiomodulation
TCM: traditional Chinese medicine
GEO: gene expression omnibus
AMP-AD: accelerating medicines partnership—Alzheimer’s disease
PPMI: Parkinson’s progression markers initiative
ZINC: ZINC is not commercial (database of commercially available compounds)
DCDB: drug combination database
NCI-ALMANAC: national cancer institute almanac (a large matrix of anti-neoplastic agent combinations)
DREAM-AZ: Dialogue for reverse engineering assessments and methods—Alzheimer’s disease
ADDI: Alzheimer’s disease data initiative
OASIS: open access series of imaging studies
MCSA: mayo clinic study of aging
AD: Alzheimer’s disease
HD: Huntington’s disease
ALS: amyotrophic lateral sclerosis
ML: machine learning
CADD: computer-aided drug design
DeepCE: deep compound embedding
GANs: generative adversarial networks
LR: logistic regression
DT: decision trees
NB: naïve bayes
KMA: K-means
AE: autoencoders
DNN: deep neural networks
RNN: recurrent neural networks
GCNs: graph convolutional networks
LBVS: ligand-based virtual screening
MTDLs: multi-target directed ligands
DOTA: drug repositioning via optimal transport for AD
PBM: photobiomodulation
BDNF: brain-derived neurotrophic factor
ITCM: integrative traditional Chinese medicine
TCGA: the cancer genome atlas
ROS/MAP: religious orders study and memory and aging project
ChEMBL: chemical database of bioactive molecules
DrugBank: a comprehensive drug and drug target database
FDA: food and drug administration
ONEIL: O’neil drug combination data set
ADNI: Alzheimer’s disease neuroimaging initiative
ADNP: activity-dependent neuroprotective protein
HABS: Harvard aging brain study
